# Oscillatory characteristics of resting-state magnetoencephalography reflect pathological and symptomatic conditions of cognitive impairment

**DOI:** 10.3389/fnagi.2024.1273738

**Published:** 2024-01-30

**Authors:** Hideyuki Hoshi, Yoko Hirata, Keisuke Fukasawa, Momoko Kobayashi, Yoshihito Shigihara

**Affiliations:** ^1^Precision Medicine Centre, Hokuto Hospital, Obihiro, Japan; ^2^Department of Neurosurgery, Kumagaya General Hospital, Kumagaya, Japan; ^3^Clinical Laboratory, Kumagaya General Hospital, Kumagaya, Japan; ^4^Precision Medicine Centre, Kumagaya General Hospital, Kumagaya, Japan

**Keywords:** magnetoencephalography, cognitive impairments, clinical neurophysiology, neurology, single-photon emission computed tomography

## Abstract

**Background:**

Dementia and mild cognitive impairment are characterised by symptoms of cognitive decline, which are typically assessed using neuropsychological assessments (NPAs), such as the Mini-Mental State Examination (MMSE) and Frontal Assessment Battery (FAB). Magnetoencephalography (MEG) is a novel clinical assessment technique that measures brain activities (summarised as oscillatory parameters), which are associated with symptoms of cognitive impairment. However, the relevance of MEG and regional cerebral blood flow (rCBF) data obtained using single-photon emission computed tomography (SPECT) has not been examined using clinical datasets. Therefore, this study aimed to investigate the relationships among MEG oscillatory parameters, clinically validated biomarkers computed from rCBF, and NPAs using outpatient data retrieved from hospital records.

**Methods:**

Clinical data from 64 individuals with mixed pathological backgrounds were retrieved and analysed. MEG oscillatory parameters, including relative power (RP) from delta to high gamma bands, mean frequency, individual alpha frequency, and Shannon’s spectral entropy, were computed for each cortical region. For SPECT data, three pathological parameters—‘*severity*’, ‘*extent*’, and ‘*ratio*’—were computed using an easy z-score imaging system (eZIS). As for NPAs, the MMSE and FAB scores were retrieved.

**Results:**

MEG oscillatory parameters were correlated with eZIS parameters. The eZIS parameters associated with Alzheimer’s disease pathology were reflected in theta power augmentation and slower shift of the alpha peak. Moreover, MEG oscillatory parameters were found to reflect NPAs. Global slowing and loss of diversity in neural oscillatory components correlated with MMSE and FAB scores, whereas the associations between eZIS parameters and NPAs were sparse.

**Conclusion:**

MEG oscillatory parameters correlated with both SPECT (i.e. eZIS) parameters and NPAs, supporting the clinical validity of MEG oscillatory parameters as pathological and symptomatic indicators. The findings indicate that various components of MEG oscillatory characteristics can provide valuable pathological and symptomatic information, making MEG data a rich resource for clinical examinations of patients with cognitive impairments. SPECT (i.e. eZIS) parameters showed no correlations with NPAs. The results contributed to a better understanding of the characteristics of electrophysiological and pathological examinations for patients with cognitive impairments, which will help to facilitate their co-use in clinical application, thereby improving patient care.

## 1 Introduction

Dementia and related cognitive impairments, such as mild cognitive impairment (MCI), remain major challenges in medicine due to neurological diseases such as Alzheimer’s disease (AD), neurovascular diseases (e.g. stroke and cerebral hemorrhage), and hydrocephalus (Elahi and Miller, 2017). Cognitive impairments and neurological diseases have a cause-effect relationship, where cognitive impairments (i.e. symptoms) are attributed to neurological diseases (i.e. pathologies). However, this relationship is nonlinear; for example, patients with AD do not consistently exhibit cognitive impairment (Snowdon, 1997; Shiroky et al., 2007). Therefore, these distinct concepts should be evaluated separately. Assessing the severity and subtyping of cognitive impairments (i.e. symptoms) is essential in the clinical treatment of dementia and MCI given that these diseases are defined by their symptoms (American Psychiatric Association, 2013; Sachdev et al., 2014), not causative pathologies, and are primarily assessed using neuropsychological assessments (NPAs) such as the Mini-Mental State Examination (MMSE) (Folstein et al., 1975; Shigemori et al., 2010) and Frontal Assessment Battery (FAB) (Dubois et al., 2000). Although these assessments are well-established and validated, they have inherent drawbacks, such as objectivity, test security, and dependency on the examinees’ background and experience (Heilbronner, 2007). In particular, practice effects interfere with the accuracy of long-term monitoring and follow-up of patients’ neurocognitive status (Wahlstrom and Boersma, 1968; Galasko et al., 1993; McCaffrey and Westervelt, 1995; Calamia et al., 2012).

Magnetoencephalography (MEG) and electroencephalography (EEG) are new clinical examination tools for supporting NPAs (Vecchio et al., 2013; Engels et al., 2017; Malek et al., 2017; López-Sanz et al., 2018; Mandal et al., 2018; Maestú et al., 2019; Giustiniani et al., 2023). MEG measures the magnetic fields generated by cortical neuronal/synaptic activity, a direct indication of brain activity (Hari and Puce, 2017; Hari et al., 2018). Patients with cognitive impairment show unique brain activity, including changes in oscillatory characteristics, based on resting-state MEG data. The MEG oscillatory characteristics of patients with cognitive impairment include (i) enhanced low-frequency oscillatory activity accompanied by attenuated high-frequency oscillatory activity, (ii) slowing down of the alpha peak frequency, (iii) less prominent alpha oscillations, and (iv) loss of diversity of neural oscillatory components (Poza et al., 2007; Fernández et al., 2013; López et al., 2014). These features are summarised as clinical parameters, such as mean frequency (MF), individual alpha frequency (IAF), Shannon’s spectral entropy (SSE), and relative power (RP), which have been used in clinical practice at our memory clinics (Hoshi et al., 2022; Hirata et al., 2024). Changes in MEG oscillatory parameters serve as direct measures of modifications in the neuronal or synaptic activities of the brain and are, therefore, strongly associated with cognitive impairment (i.e. cognitive symptoms) (Hoshi et al., 2022). Furthermore, MEG oscillatory parameters are advantageous in their clinical use as they require low computational costs (e.g. the calculation requires approximately 10 min using a laptop computer).

Single-photon emission computed tomography (SPECT) has been used in clinical practice for more than 20 years (Jagust et al., 1995; Davis et al., 2020) to assess the level of perfusion by measuring the regional cerebral blood flow (rCBF), which is affected by various pathological conditions, such as AD (Albert et al., 2011) and dementia with Lewy bodies (DLB) (McKeith et al., 2017). Analysis pipelines, such as the ‘easy Z-score imaging system (eZIS)’, are well established. The eZIS parameters are a set of biomarkers that indicate the probability of AD (Matsuda et al., 2007) or DLB (Imabayashi et al., 2017) and parameterise changes in the rCBF, which are affected by the pathological conditions of these diseases. The levels of hypoperfusion and hypometabolism are midstream pathological biomarkers of AD (Bjorkli et al., 2020), reflecting the synaptic dysfunction caused by these diseases (Fagan, 2014). Based on the assumption of neurovascular coupling (Iadecola, 2017), rCBF is considered an indirect measurement of neural activity. Therefore, there is an association between MEG/EEG oscillatory parameters (i.e. direct measurements of synaptic dysfunction) and eZIS parameters (i.e. measurements of rCBF/hypoperfusion and clinically validated midstream biomarkers). No prior research has explored the correlation between MEG oscillatory and eZIS parameters. When incorporating MEG and eZIS parameters into clinical practice, it is crucial to accurately grasp both their commonalities and distinctions.

Therefore, in this study, we investigated the relationship between MEG oscillatory and eZIS parameters using actual clinical data obtained retrospectively from the clinical records of individuals who visited the outpatient department of dementia at our hospital. Additionally, to examine the relevance to clinical symptoms, two NPA scores, the MMSE and FAB, were retrieved from the records, and their relationships with MEG oscillatory and eZIS parameters were assessed. We hypothesise that MEG oscillatory parameters are associated with both pathological and symptomatic changes and that their symptomatic associations are stronger than those of the eZIS parameters since eZIS parameters are indirect measurements of neural activity (i.e. neurovascular coupling). According to this hypothesis, we expected that MEG oscillatory parameters would be correlated with both eZIS parameters and NPAs, whereas the direct relationships between eZIS parameters and NPAs would be sparse. A better understanding of the similarities and differences between MEG oscillatory and eZIS parameters will help to facilitate their clinical application, thereby improving patient care.

## 2 Materials and methods

### 2.1 Participants and ethics

The clinical records of 72 individuals who visited the outpatient department of Kumagaya General Hospital (Saitama, Japan) were retrieved. We set rough exclusion criteria to align the background of individuals in this study as closely as possible with that of the clinical population in the outpatient department. This led to the exclusion of (1) one individual with a non-dementia disease, (2) four individuals who were undiagnosed at the time of examination, and (3) three individuals whose MEG data were contaminated from severe artifacts. Consequently, data from the remaining 64 individuals were included in the analysis (35 females, mean age ± standard deviation: 77.0 ± 7.0 years; age range: 53–91 years; Table 1). The retrieved data included MEG data, eZIS parameters computed from the SPECT measurements, and NPAs. Among the 64 individuals, 9 had healthy ageing, 15 were diagnosed with MCI, 30 with AD, and 10 with other types of dementia [i.e. vascular dementia (VD), DLB, frontotemporal dementia (FTD), or a combination of them]. Detailed information on each diagnostic group is shown in Supplementary Table S1. This study was conducted in accordance with the ethical principles of the Declaration of Helsinki and was approved by the Ethics Committee of Kumagaya General Hospital (approval number: #76). All individuals provided written informed consent to participate in this study if they were cognitively healthy. Otherwise, their legal guardians/next of kin provided consent on their behalf.

**Table 1:** Descriptive statistics.

		*M*	SD	MIN	MAX
	Age	76.95	6.977	53	91
NPAs	MMSE	24.16	5.109	9	30
	FAB	11.97	3.137	4	18
eZIS	*severity*	1.299	0.495	0.680	3.170
	*extent*	15.84	14.80	0.760	64.62
	*ratio*	2.109	1.615	0.060	6.130
MEG	RPd	0.192	0.067	0.064	0.339
	RPt	0.124	0.045	0.045	0.280
	RPa1	0.112	0.043	0.034	0.231
	RPa2	0.110	0.046	0.044	0.286
	RPa3	0.063	0.021	0.031	0.141
	RPb	0.218	0.068	0.073	0.450
	RPlg	0.068	0.022	0.022	0.133
	RPhg	0.052	0.019	0.018	0.108
	MF	9.425	2.120	5.650	16.68
	IAF	8.665	0.800	6.865	10.38
	SSE	0.787	0.029	0.714	0.840

M, mean; SD, standard deviation; NPAs, neuropsychological assessments; MMSE, Mini-Mental State Examination; FAB, Frontal Assessment Battery; eZIS, easy Z-score imaging system; MEG, magnetoencephalography; RPd, relative power in delta band; RPt, relative power in theta band; RPa1, relative power in alpha1 band; RPa2, relative power in alpha2 band; RPa3, relative power in alpha3 band; RPb, relative power in beta band; RPlg, relative power in low gamma band; RPhg, relative power in high gamma band; MF, mean frequency; IAF, individual alpha frequency; SSE, Shannon’s spectral entropy.

### 2.2 Neuropsychological assessments

For the NPAs, scores from two assessments, namely MMSE and FAB, were retrieved. These assessments are scored on scales of 0–30 and 0–18, respectively, where lower scores indicate more severe cognitive impairment. MMSE is the most commonly used assessment tool for dementia screening (Shigemori et al., 2010) and primarily evaluates learning/memory performance (Dinomais et al., 2016). In contrast, FAB assesses executive functions, including attention, inhibition, working memory, interference control, and cognitive flexibility (Diamond, 2013), which are served by the prefrontal cortex (Gilbert and Burgess, 2008). The assessments were administered by clinical psychologists as part of clinical practice. Notably, FAB was not administered to an individual who did not wish to be assessed; thus, all statistical analyses involving FAB data were performed using data from 63 individuals (out of 64 individuals). The descriptive statistics of the NPAs are summarised in Table 1.

### 2.3 SPECT measurement and data analysis

To assess the pathological conditions, we used three parameters—namely ‘*severity*’, ‘*extent*’, and ‘*ratio*’—determined using the eZIS software (PDRadiopharma Inc., Tokyo, Japan). These parameters were based on rCBF images obtained through SPECT (Mizumura and Kumita, 2006; Matsuda et al., 2007), which are sensitive to pathological changes in conditions such as AD. SPECT scanning was performed as part of the clinical assessment, and the scores were retrieved from the clinical records. Prior to SPECT measurement, all participants received an intravenous injection of 600 MBq of 99mTc-ethylcysteinate dimer (PDRadiopharma Inc. Tokyo, Japan). After 5 min of rest with eyes closed in the supine position in a dark room, SPECT data were obtained using a 128 × 128 matrix on a BrightView X (Philips Healthcare, Milpitas, CA, United States) equipped with a low-energy, cardiac high-resolution parallel-hole collimator and dual thallium-activated sodium iodide scintillation detector. Seventy-two views were obtained continuously throughout the 360° rotation (5°/step, zoom 1.85) with a pixel size of 3.2 mm. The acquired images were reconstructed using the filtered back-projection method with combined Chang attenuation correction. The reconstructed rCBF images were analysed using eZIS, which implemented the Statistical Parametric Mapping 2 toolbox (Wellcome Trust Centre for Neuroimaging, London, UK^1^) and automated image processing, including spatial normalisation, smoothing, and Z-scoring, by comparing the data against an age-matched control database. In the eZIS software, the Z-score was obtained as [(control mean) – (individual value)]/(control SD), where a higher Z-score indicates more reduction in rCBF and severe hypoperfusion (Matsuda et al., 2007). The Z-scored images were summarised within a pre-defined volume of interest (VOI) set bilaterally on the posterior cingulate gyrus (PCG), precuneus (PC), and parietal lobe. This selection was based on a previous study that demonstrated group-level differences in rCBF images between patients with amnestic MCI (aMCI) due to AD and individuals with healthy ageing (Matsuda et al., 2007). Notably, although the cortical regions (PCG, PC, and parietal lobe) were spatially discontinuous, they were considered a single VOI. Three primary parameters were computed using the Z-score summarised in the VOI: ‘*severity*,’ ‘*extent*’, and ‘*ratio*’ (Matsuda et al., 2007; Hayashi et al., 2020). The *severity* indicated the degree of decrease in the average Z-scored rCBF in the VOI. The *extent* indicated the percentage of voxels with a Z-score > 2 relative to the total number of voxels in the VOI. The *ratio* indicated the specificity of rCBF reduction in the VOI, which is the rate of the *extent* value to that computed using the whole-brain volume instead of the VOI. For the three parameters, higher values corresponded to more severe hypoperfusion of the VOI. To discriminate patients with aMCI due to AD from individuals with healthy ageing, optimal cut-off thresholds were set at 1.19, 14.2, and 2.22 for *severity*, *extent*, and *ratio*, respectively (Matsuda et al., 2007). The descriptive statistics of the eZIS parameters are summarised in Table 1.

### 2.4 MEG measurement

Resting-state MEG data were collected from clinical records. The resting-state cortical activity was recorded for 5 min using a whole-head-type MEG system (RICOH160-1; RICOH Co. Ltd., Tokyo, Japan) equipped with 160-channel axial gradiometers and placed in a magnetically shielded room at Kumagaya General Hospital. During the scan, the participants were asked to remain awake and calm in a supine position with their eyes closed. The sensor coils were gradiometers with diameters of 15 mm and heights of 50 mm. The pairs of sensor coils were separated 23 mm apart. The sampling frequency was 2,000 Hz with 500-Hz low-pass filtering during recording. To co-register the MEG data with the anatomical brain images, five fiducial magnetic marker coils were placed on each participant’s face (40 mm above the nasion, bilaterally 10 mm in front of the tragus, and at the bilateral pre-auricular points) prior to the MEG scan. The spatial coordinates of these markers were measured immediately before scanning. To maintain the optimal state of vigilance, the recording was initiated shortly after closing the door of the magnetically shielded room. Participants were also reminded to stay awake with their eyes closed via the intercom prior to scanning, if necessary. During the scan, participants were monitored by medical technicians using a video camera installed in a magnetically shielded room, and their vigilance states were verified using self-reports collected after the scan.

### 2.5 MEG data analysis

MEG data were analysed offline using the RICOH MEG Analysis software (RICOH, Tokyo, Japan), MATLAB (MathWorks, MA, United States), and Brainstorm, which is documented and freely available for download online under the GNU general public license^2^ (Tadel et al., 2011). The pre-processing pipeline followed the strategy used in a previous study (Rodríguez-González et al., 2020). First, continuous MEG signals were cleaned using a dual-signal subspace projection algorithm (Sekihara et al., 2016) available on vendor-provided software (RICOH MEG Analysis), which is comparable to the temporally extended signal space separation algorithm, with the only difference being the approximation of the signal subspace projector (Cai et al., 2019). Next, to remove the remaining artifacts, the signals were decomposed with independent component analysis (ICA) using the FastICA algorithm implemented in Brainstorm (Makeig et al., 1996). Each ICA component was visually inspected, and those for cardiac, blinking, and other salient artifacts were rejected. Artifact-free signals were filtered using Finite Impulse Response Filtering with a Hamming window by applying a bandpass (1–70 Hz) to limit the noise bandwidth and a bandstop (48–52 Hz) to remove line noise.

The filtered signals were then imported to Brainstorm, where they were projected onto the cortical source using the software’s default parameters. ICBM152, a template anatomical brain image prepared by Brainstorm, was used for the analysis. ICBM152 is a nonlinear average of 152 magnetic resonance images from different subjects (Fonov et al., 2009) and is provided along with its cortical segments. The signal source was restricted to the cortex, which was segmented into 15,000 vertices. Each MEG data set was co-registered with the anatomical image using the spatial coordinates of five fiducial points and the nasion, and the relationship between 160 MEG channels and 15,000 vertices (i.e. leadfield matrix) was modeled (i.e. forward modeling) using a Symmetric Boundary Element Method (Kybic et al., 2005; Gramfort et al., 2010), which generated a three-layer (brain, skull, and scalp) realistic head model. Prior to computing the source signals, the characteristics of the MEG sensor noise were modeled as a covariance matrix for each pair of channels (i.e. noise covariance), which was defined as the average of the covariance matrices across eight empty-room recordings (5 min) measured using an identical MEG machine with the same acquisition setting. Using the forward model and noise covariance matrix, the source signals of MEG data were computed using the Weighted Minimum Norm Estimation (wMNE) method (Lin et al., 2006). The wMNE restricts the sources of the inverse problem by minimising the energy (L2 norm) while weighting the deep sources to facilitate their detection. This algorithm was selected for three reasons: (i) it is recommended as a default option in Brainstorm, (ii) we utilised template brain instead of individual MRI images, which provides only rough approximations of the forward model and is unsuitable for other inversion algorithms (e.g. Beamforming) requiring better model approximation than wMNE, and (iii) wMNE is widely used in the context of MEG and EEG source localization for studying oscillatory characteristics of pathological conditions (Larson-Prior et al., 2013; Rizkallah et al., 2020; Rodríguez-González et al., 2020; Tait et al., 2021). The orientation of the neural sources was restricted to be normal to the cortex. The resulting data from the source reconstruction process were continuous time-series signals for each of the 15,000 cortical vertices. High-dimensional data were limited to 103 anatomical regions defined by the Automated Anatomical Labeling Atlas 3 (AAL3) (Rolls et al., 2020) by averaging the signals of the vertices included in each anatomical region. We used the AAL3 atlas because it corresponds to the atlas used for labeling the VOI in eZIS (Imabayashi et al., 2016, 2017). During the averaging process, the signs of the signals were flipped in the vertices, where the normal orientation was opposite to the dominant orientation of the corresponding region.

To extract the oscillatory power from regional time-series signals, the power spectral density (PSD) was computed using the Blackman-Tukey approach (Blackman and Tukey, 1958) with non-overlapping 5-s segments. In the Blackman-Tukey method, PSD is defined as a discrete Fourier transform of the autocorrelation function of the time-series data, which has better precision than other approaches (Kale et al., 2013) and is commonly used for computing MEG oscillatory parameters (Poza et al., 2007, 2008a,b; Gómez et al., 2013; Rodríguez-González et al., 2020; Hoshi et al., 2022). To obtain the normalised PSD (PSDn), the original PSD was divided by the total power in the frequency range of interest, i.e. 1–70 Hz (Gómez et al., 2013). Next, the RP in each canonical frequency band [delta (1–3 Hz), theta (4–7 Hz), alpha1 (7–9 Hz), alpha2 (9–11 Hz), alpha3 (11–13 Hz), beta (13–25 Hz), and gamma (low gamma, 26–40 Hz; high gamma, 41–70 Hz)] was computed by calculating the cumulative sum of the power. The RP in each band was referred to as RPd (delta), RPt (theta), RPa1 (alpha1), RPa2 (alpha2), RPa3 (alpha3), RPb (beta), RPlg (low gamma), and RPhg (high gamma). Notably, the alpha band was subdivided into three sub-bands (alpha1, alpha2, and alpha3) because their correlational behaviours to the level of cognitive impairments were expected to be opposite before and after the alpha peak (Poza et al., 2007; Gómez et al., 2013; López et al., 2014), which would cancel out if the bands were considered as whole. Previous studies proposed flexible and individually adjusted definitions of frequency bands (Babiloni et al., 2020), including three alpha sub-bands (Klimesch, 1999; Doppelmayr et al., 2002); however, this study focused on the existing MEG oscillatory parameters, which are defined using fixed canonical frequency bands. Hence, the canonical alpha sub-bands were set following a previous study (Wu and Liu, 1995). Finally, three spectral parameters were calculated to summarise the different properties of PSDn: MF, IAF, and SSE (Poza et al., 2008b), the definitions and details of which have been previously reported (Poza et al., 2007). The first parameter, MF, quantifies the frequency at which the spectral power is balanced between low and high frequencies. The frequency divides PSDn into two halves, 1 Hz and 70 Hz. The second parameter, IAF, represents the dominant frequency corresponding to the peak of the PSDn in the alpha band and is defined similarly to MF, except that the frequency range is adjusted to 4–15 Hz (i.e. extended alpha band), to obtain a robust estimator of the dominant alpha oscillations (Poza et al., 2007). The last parameter, SSE, is defined by applying the definition of the normalised Shannon entropy to PSDn, which can be assimilated as a probability density function (Poza et al., 2007):


$$\mathrm{SSE}=-\frac{1}{\log\left(N\right)}\cdot \overset{70Hz}{\underset{f=1Hz}{\sum }}PSDn\left(f\right)\log\left[PSDn\left(f\right)\right]$$

Where N is the number of frequency bins of PSDn. SSE represents an irregularity measure closely related to the concept of order in information theory, which quantifies the homogeneity in the distribution of the oscillatory components of the PSDn. All spectral parameters (MF, IAF, and SSE) have been considered as reflections of the levels of cognitive impairments (Engels et al., 2017; López-Sanz et al., 2018; Mandal et al., 2018; Maestú et al., 2019), presumably capturing different aspects of the cognitive impairments. This interpretation is supported by a previous study that demonstrated the relationships between MF and MMSE/FAB, IAF and MMSE, and SSE and FAB (Hoshi et al., 2022). Therefore, in this study, the three parameters were considered separately. Region- and epoch-wise RPs, MF, IAF, and SSE, were computed, averaged across epochs, and used in the statistical analysis. Descriptive statistics of the average MEG oscillatory parameters are summarised in Table 1. The regional distribution of the MEG oscillatory parameters is shown in Supplementary Figure S1.

### 2.6 Statistical analysis

Statistical analyses were performed using MATLAB (MathWorks, MA, United States), the Fieldtrip toolbox (Maris and Oostenveld, 2007; Oostenveld et al., 2011), the Statistics and Machine Learning Toolbox (MathWorks, Natick, MA, United States), and the Multiple Testing Toolbox (Martínez-Cagigal, 2021).

In previous studies, the MEG oscillatory parameters were first examined by summarising across all sensors (Fernández et al., 2006; Poza et al., 2007, 2008a,b; Gómez et al., 2013), and their global changes were considered as clinical parameters for capturing cognitive impairments (Hoshi et al., 2022). Therefore, first, the global relationships between MEG oscillatory parameters (RPs in eight canonical frequency bands, MF, IAF, and SSE) averaged across anatomical regions, three eZIS parameters (*severity*, *extent*, and *ratio*), two NPA scores (MMSE and FAB), and the age of the individuals were assessed. For each pair of parameters, a 95% bootstrap confidence interval of Spearman’s coefficient (*rho*) was computed by 5,000 bootstrap iterations, and the correlation was considered significant when the interval did not contain 0. The within-modality correlations among MEG oscillatory or eZIS parameters (e.g. RPd × RPt, *severity* × *extent*) were not examined because they are not within the scope of this study. Moreover, two biasing factors exist for the within-modality correlations of MEG oscillatory parameters: 1/f activity and normalisation. The changes in slope (i.e. steepness) of 1/f activity, a broadband pattern underlying the PSDn, lead to rotational reshaping of PSDn, resulting in antagonistic behaviours between low- and high-frequency bands. Additionally, changes in intercept (i.e. offset) contribute to overall increasing/decreasing of the PSDn, causing sympathetic behaviours between low- and high-frequency bands. The normalisation converts the absolute power to relative power across frequency bins, creating a pseudo trade-off relationship between them. Due to their intercorrelating nature in the definition, we deemed evaluations of within-modality correlations not meaningful. Notably, the nonlinear Spearman’s correlation is robust for the outliers (de Winter et al., 2016). Therefore, we did not screen out the outliers in the MEG oscillatory parameters but instead removed the dataset by inspecting the raw MEG signals (see Section 2.1).

Next, the regional relationships between the MEG oscillatory parameters, three eZIS parameters, and two NPA scores were examined using a non-parametric cluster-based permutation approach implemented in the Fieldtrip toolbox (Maris and Oostenveld, 2007). The non-parametric cluster-based permutation approach was used for controlling family-wise error rate in multiple comparisons. This approach empirically identifies ‘clusters’, encompassing multiple adjacent regions where the neuroimaging data exhibit similar behaviours concerning the effect of interest (*T*-statistic). As a first step of the permutation test, for each pair of MEG oscillatory parameters and eZIS parameters or NPA scores, the *T*-statistic of Spearman’s coefficient (*rho*) was calculated in the region (103) space (observed statistics) using a function implemented in the Fieldtrip toolbox (ft_statfun_correlationT.m) with a following formula:


$$T=\frac{rho\sqrt{\left(N-2\right)}}{\sqrt{1-rho^{2}}}$$

Where N is the number of observations (individuals). The *T*-statistic was also computed using a shuffled dataset across participants (e.g. the MEG parameter for participant A was paired with the eZIS parameter of participant B) 5,000 times, which generated a probability distribution of the random statistics. For each region, the observed statistics were examined if they were above or below the critical value (0.025) in the right or left tail of the random probability distribution, respectively. The regions where the observed statistics exceeded the tails were clustered according to their spatial adjacency on each side of the tail and defined as positive and negative clusters. The observed *T*-statistic was summed in each cluster (observed cluster statistics), while the random distribution of cluster statistics was generated by collecting the maximum of summed *T*-statistics among detected clusters in the shuffled data for each of the 5,000 iterations (‘maxsum’ method in cluster-based permutation algorithm in the Fieldtrip toolbox). The percentage of random cluster statistics that were larger (for positive clusters) or smaller (for negative clusters) than the observed cluster statistics was considered as the significance level (*p*-value) of each observed cluster. We reported the average *rho* [*rho* (mean)] and average *T*-statistics [*T* (mean)] across regions included in each cluster, and corresponding *p*-value. Notably, while the cluster-based permutation approach addressed the multiple comparison problem between multiple regions, we did not address the problem between multiple pairs of the cluster-based permutation tests (i.e. repeated use of the tests). This decision was based on the reason that the analyses were exploratory; regarding the MEG oscillatory and eZIS parameters, lacking a priori hypotheses and focusing on specific parameters.

The potential confounding effects of age were examined in the final step of the cluster-based permutation test. As the global correlation analysis revealed that age correlated with the *ratio* of eZIS parameters and the two NPA scores (i.e. MMSE and FAB), their relationships with MEG oscillatory parameters could be confounded by age (i.e. pseudo-correlations). To examine this, the contributions of the three variables (*ratio*, MMSE, or FAB) and age to the MEG oscillatory parameters were evaluated using the partial least squares (PLS) regression. Taking each MEG oscillatory parameter in each region as a response variable, PLS regression was performed using two predictors, namely one of the three variables and age, with a one-component model. The variable importance in projection (VIP) scores were computed for each predictor and compared for each MEG oscillatory parameter in every region. Larger VIP scores for age compared to those for one of the other variables (*ratio*, MMSE, and FAB) indicated that the contribution of age was stronger to the regional MEG oscillatory parameter than the variable. In such cases, we considered the possibility of potential confounding effects of age on the relationship between the variable and the regional MEG oscillatory parameter. Therefore, the region was excluded from the significant clusters found in the cluster-based permutation tests for the variable.

To aid the interpretation of the results, statistical analyses were repeated using data from patients with MCI and AD (*N* = 45), presented in Supplementary sections B–D.

## 3 Results

### 3.1 Global correlations between MEG oscillatory parameters, eZIS parameters, and NPAs

**Figure 1:**
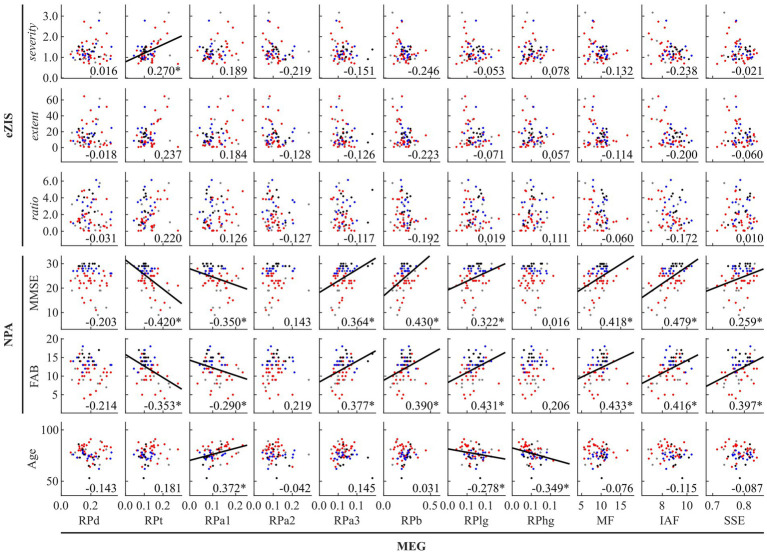
Between-modality correlations between MEG oscillatory parameters averaged across anatomical regions, eZIS parameters, NPAs, and age. The scatterplot is colored differently for each diagnosis (black: healthy ageing, blue: MCI, red: AD, and grey: other types of dementia). Regression lines are added for significant correlations. The number displayed at the corner of each plot indicates Spearman’s coefficient (*rho*) averaged across bootstrap iterations, with an asterisk (*) indicating significant correlation. eZIS, easy Z-score imaging system; NPAs, neuropsychological assessments; MMSE, Mini-Mental State Examination; FAB, Frontal Assessment Battery; MEG, magnetoencephalography; RPd, relative power in delta band; RPt, relative power in theta band; RPa1, relative power in alpha1 band; RPa2, relative power in alpha2 band; RPa3, relative power in alpha3 band; RPb, relative power in beta band; RPlg, relative power in low gamma band; RPhg, relative power in high gamma band; MF, mean frequency; IAF, individual alpha frequency; SSE, Shannon’s spectral entropy.

**Figure 2:**
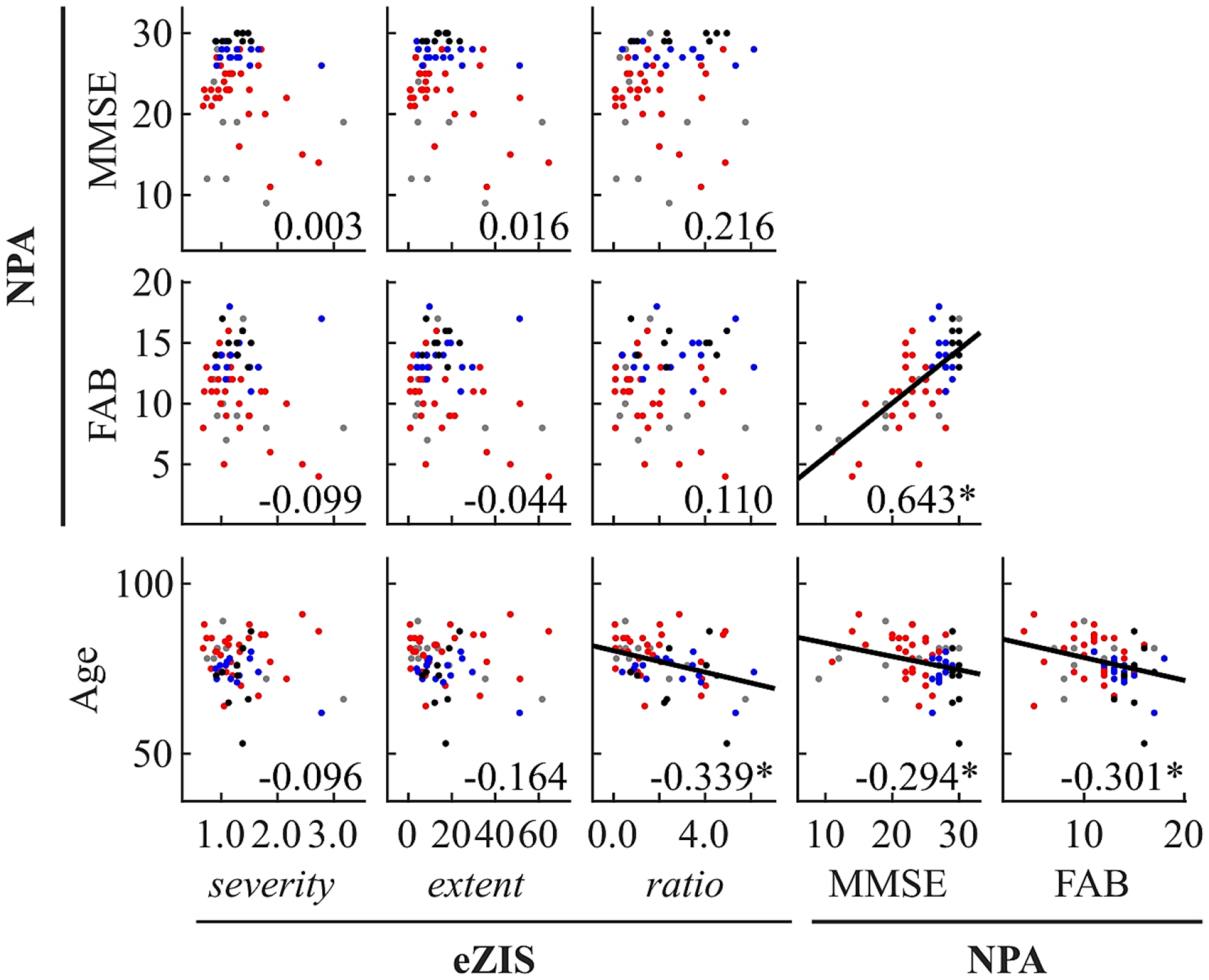
Within- and between-modality correlations among eZIS parameters, NPAs, and age. Regression lines are added for significant correlations. The scatterplot is colored differently for each diagnosis (black: healthy ageing, blue: MCI, red: AD, and grey: other types of dementia). The number displayed at the corner of each plot indicates Spearman’s coefficient (*rho*) averaged across bootstrap iterations, with an asterisk (*) indicating significant correlation. eZIS, easy Z-score imaging system; NPAs, neuropsychological assessments; MMSE, Mini-Mental State Examination; FAB, Frontal Assessment Battery.

The results of the global correlations between the MEG oscillatory parameters, eZIS parameters, NPAs, and age are illustrated in Figure 1 (MEG vs. the others), Figure 2 (between the others), and Supplementary Tables S7, S8.

Figure 1 and Supplementary Table S7 summarise the between-modality correlations of MEG parameters with eZIS parameters, NPAs, and individuals’ age. Among the MEG × eZIS parameters, RPt was positively correlated with *severity*. For MEG × NPAs, the RPs in slower bands (RPt and RPa1) showed constant negative correlations with NPAs, whereas the RPs in faster bands (RPa3, RPb, and RPlg) showed positive correlations. Moreover, the MEG spectral parameters also showed positive correlations with NPAs. For MEG × age, it was only sparsely correlated with the MEG oscillatory parameters of RPa1, RPlg, and RPhg. No other pair showed significant global correlations.

Figure 2 and Supplementary Table S8 summarise the results of between-modality correlations of eZIS parameters, NPAs, and age. The eZIS parameters did not correlate with NPAs, while the *ratio* was correlated negatively with age. The NPAs showed strong positive intercorrelations between MMSE and FAB and negative correlations with age.

### 3.2 Regional correlations between MEG oscillatory and eZIS parameters

**Figure 3:**
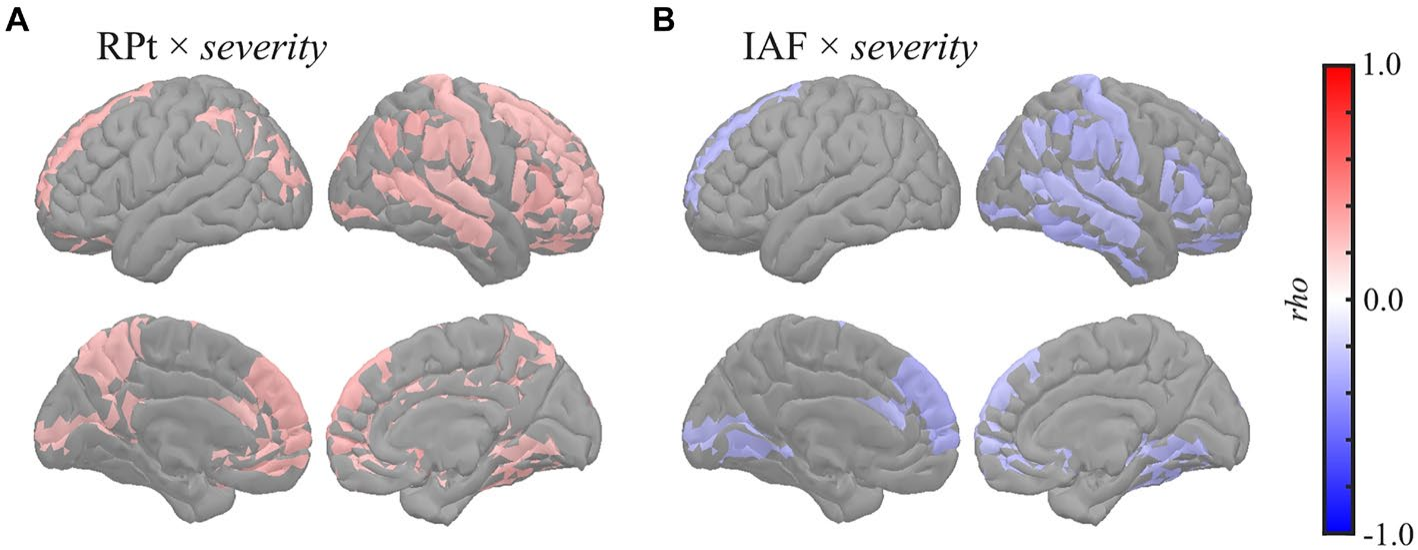
Regional correlations between MEG oscillatory and eZIS parameters. Cortical projection of significant clusters (*p* < 0.025), indicated by colored regions with correlation coefficients between **(A)** RPt and *severity* and **(B)** IAF and *severity*. *rho*, Spearman’s coefficient; MEG, magnetoencephalography; RPt, relative power in theta band; IAF, individual alpha frequency; eZIS, easy Z-score imaging system.

Global correlation analysis demonstrated that the average MEG oscillatory parameters across all regions were associated with the eZIS parameters. To investigate the cortical regions involved in these associations, we conducted regional analyses of the relationships using cluster-based permutation tests in the region (103) space (Figure 3). RPt showed positive correlations with *severity* [*rho* (mean) = 0.281, *T* (mean) = 2.310, *p* = 0.012] in globally distributed regions, particularly prominent in the right hemisphere (Figure 3A). Similarly, IAF demonstrated negative correlations with *severity* [*rho* (mean) = −0.279, *T* (mean) = −2.290, *p* = 0.022] in globally distributed regions, which was also more prominent in the right hemisphere (Figure 3B). The regions included in the clusters are listed in Supplementary Table S9. No significant clusters were observed for the correlations between other pairs of MEG oscillatory parameters and eZIS parameters.

### 3.3 Regional correlations between MEG oscillatory parameters and NPAs

**Figure 4:**
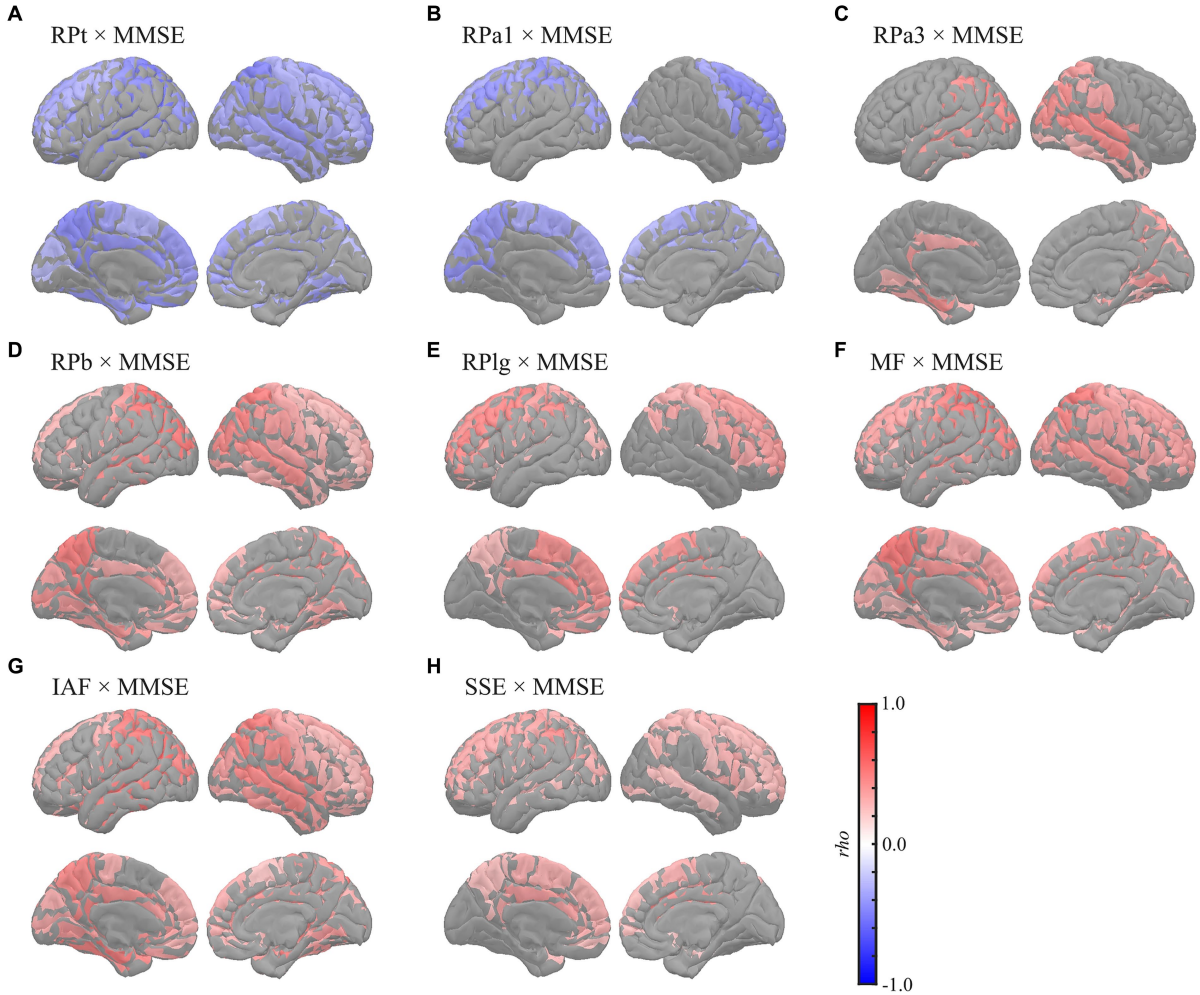
Regional correlations between MEG oscillatory parameters and MMSE. Cortical projection of significant clusters (*p* < 0.025), indicated by colored regions with correlation coefficients between MMSE and **(A)** RPt, **(B)** RPa1, **(C)** RPa3, **(D)** RPb, **(E)** RPlg, **(F)** MF, **(G)** IAF, and **(H)** SSE. *rho*, Spearman’s coefficient; MEG, magnetoencephalography; RPt, relative power in theta band; RPa1, relative power in alpha1 band; RPa3, relative power in alpha3 band; RPb, relative power in beta band; RPlg, relative power in low gamma band; MF, mean frequency; IAF, individual alpha frequency; SSE, Shannon’s spectral entropy; MMSE, Mini-Mental State Examination.

**Figure 5:**
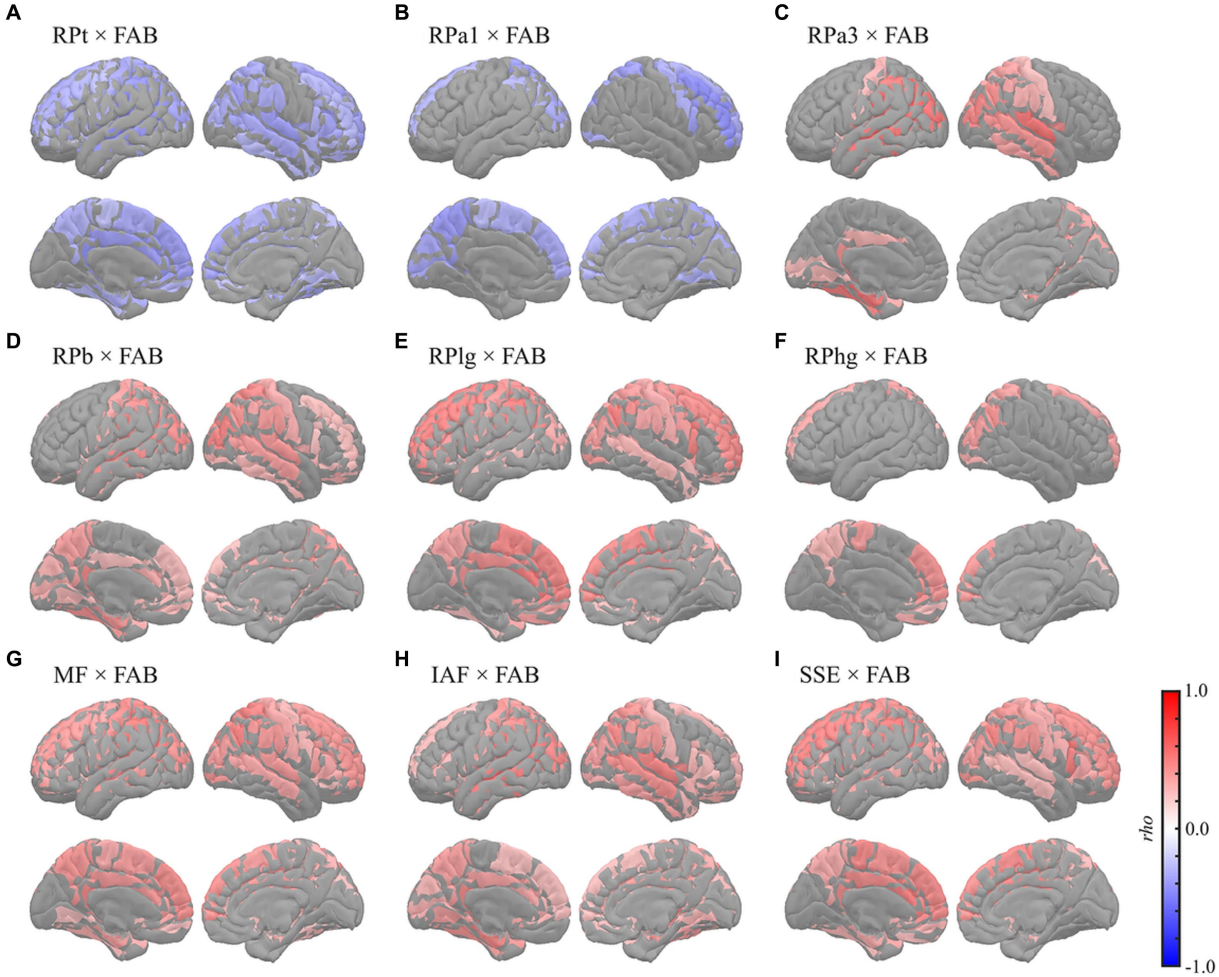
Regional correlations between MEG oscillatory parameters and FAB. Cortical projection of significant clusters (*p* < 0.025), indicated by colored regions with correlation coefficients between FAB and **(A)** RPt, **(B)** RPa1, **(C)** RPa3, **(D)** RPb, **(E)** RPlg, **(F)** RPhg, **(G)** MF, **(H)** IAF, and **(I)** SSE. *rho*, Spearman’s coefficient; MEG, magnetoencephalography; RPt, relative power in theta band; band; RPa1, relative power in alpha1 band; RPa3, relative power in alpha3 band; RPb, relative power in beta band; RPlg, relative power in low gamma band; RPhg, relative power in high gamma band; MF, mean frequency; IAF, individual alpha frequency; SSE, Shannon’s spectral entropy; FAB, Frontal Assessment Battery.

Global correlation analyses demonstrated that the average MEG oscillatory parameters across all regions were associated with NPAs. To investigate the specific cortical regions involved in the associations, we conducted analyses of regional relationships using cluster-based permutation tests in the region (103) space (Figures 4, 5). MMSE (Figure 4) showed negative correlations with RPs in slower bands; global RPt [*rho* (mean) = −0.387, *T* (mean) = −3.332, *p* < 0.001] and dorsal RPa1 [*rho* (mean) = −0.374, *T* (mean) = −3.188, *p* = 0.002] (Figures 4A,B), positive correlations with faster bands; occipitotemporal RPa3 [*rho* (mean) = 0.378, *T* (mean) = 3.248, *p* = 0.003], global RPb [*rho* (mean) = 0.373, *T* (mean) = 3.204, *p* = 0.001], and dorsal RPlg [*rho* (mean) = 0.365, *T* (mean) = 3.119, *p* = 0.003] (Figures 4C–E), and positive correlations with spectral parameters; global MF [*rho* (mean) = 0.373, *T* (mean) = 3.214, *p* = 0.001], IAF [*rho* (mean) = 0.423, *T* (mean) = 3.731, *p* < 0.001], and dorsal SSE [*rho* (mean) = 0.316, *T* (mean) = 2.641, *p* = 0.003] (Figures 4F–H).

The FAB had similar regional relationships to MEG oscillatory parameters as the MMSE (Figure 5). It demonstrated negative correlations with RPs in slower bands; global RPt [*rho* (mean) = −0.345, *T* (mean) = −2.888, *p* = 0.003] and dorsal RPa1 [*rho* (mean) = −0.345, *T* (mean) = −2.879, *p* = 0.009] (Figures 5A,B), positive correlations with faster bands; occipitotemporal RPa3 [*rho* (mean) = 0.419, *T* (mean) = 3.687, *p* = 0.002], occipitotemporal RPb [*rho* (mean) = 0.351, *T* (mean) = 2.964, *p* = 0.001], dorsal RPlg [*rho* (mean) = 0.371, *T* (mean) = 3.161, *p* = 0.001], and dorsal RPhg [*rho* (mean) = 0.347, *T* (mean) = 2.906, *p* = 0.010] (Figures 5C–F), and positive correlations with spectral parameters; global MF [*rho* (mean) = 0.369, *T* (mean) = 3.138, *p* = 0.001], IAF [*rho* (mean) = 0.379, *T* (mean) = 3.243, *p* < 0.001], and dorsal SSE [*rho* (mean) = 0.379, *T* (mean) = 3.242, *p* < 0.001] (Figures 5G–I). The regions included in the clusters are listed in Supplementary Tables S10–S15. No significant clusters were observed for the correlations between the other pairs of MEG oscillatory parameters and NPAs.

## 4 Discussion

This study revealed that the MEG oscillatory parameters correlated with the eZIS parameters. Specifically, the clinical parameters related to AD pathology were linked to the theta power augmentation and slower shift of the alpha peak, predominantly observed in the right hemisphere. Furthermore, MEG oscillatory parameters were found to reflect NPAs; global slowing and loss of diversity in the neural oscillatory components were correlated with MMSE and FAB scores, whereas the associations between eZIS parameters and NPAs were less pronounced. Overall, the results largely supported our hypothesis that MEG oscillatory parameters are associated with both pathological and symptomatic changes and that their symptomatic relationships are more explicit than those of the eZIS parameters.

In this study, we focused on the MEG oscillatory parameters because they are useful in clinical examinations for supporting NPAs (Engels et al., 2017; López-Sanz et al., 2018; Mandal et al., 2018; Maestú et al., 2019). Similarly to MEG, EEG also captures electrophysiological activities, which can be summarise as oscillatory parameters and shown to be sensitive to cognitive impairment (Vecchio et al., 2013; Malek et al., 2017; Giustiniani et al., 2023). MEG and EEG each have their own set of advantages and disadvantages. From a practical viewpoint, EEG is advantageous in terms of facility accessibility (i.e. prevalence) and installation/maintenance costs. However, its preparation requires a lot of effort, incurring costs for busy clinicians. Moreover, setting up EEG involves attaching electrodes to the scalp with gel paste, requiring patients to remain seated stably, which often is uncomfortable and challenging for patients with cognitive impairment. On the other hand, while MEG is expensive and only available in limited hospitals, its preparation and measurement processes are more straightforward than those of EEG. Patients only need to lie on the bed, and examiners attach five marker coils (some MEG systems require a lengthy procedure called digitization of the head, but it is only optional for our system; RICOH 160-1). From a technical perspective, MEG avoids selection bias associated with reference electrodes, a factor that always impacts EEG data analysis. In addition, MEG is traditionally and theoretically considered more accurate in the source estimations (Cohen et al., 1990). However, the superiority of MEG is not empirically supported; direct comparisons of the source estimation accuracies between MEG and EEG (particularly high-density EEG) provided comparable results (Cohen and Cuffin, 1991; Liu et al., 2002; Hedrich et al., 2017). Furthermore, the oscillatory parameters are not different between sensor- and source-levels. The source-level oscillatory parameters, averaged within five regions of interest (left frontal, right frontal, left temporal, right temporal, and occipital), were shown to be correlated strongly with the sensor-level parameters, averaged within regions of interest with spatial topographies similar to those used at the source-level (Rodríguez-González et al., 2020). This suggests that oscillatory parameters provide equivalent information at both sensor- and source-levels, even when performing regional analyses. Consequently, any potential advantage in the source estimation accuracy of MEG, if present, may not provide additional benefits in examining patients with cognitive impairment using oscillatory parameters. Overall, both MEG and EEG have comparable performance in the clinical practice of electrophysiological examinations of patients with cognitive impairments, which are currently selected based on the preferences of researchers or practical reasons in each hospital/clinic. In this study, our focus was on MEG, not only due to our preference for its convenience and patient-friendliness, but also because recent advancements in MEG hardware (e.g. optically pumped magnetometers) are overcoming cost limitations and expanding accessibility (Brookes et al., 2022). This development encourages the active integration of MEG examinations into clinical practices in the near future.

### 4.1 Associations between MEG oscillatory and eZIS parameters

MEG has been used for the clinical examination of cognitive impairment (Hoshi et al., 2022; Hirata et al., 2024), as it provides oscillatory parameters sensitive to cognitive impairment. However, its association with pathological conditions remains controversial. According to the amyloid hypothesis of AD (Jack et al., 2010; Fagan, 2014; Bjorkli et al., 2020), neuronal/synaptic changes begin following the appearance of amyloid-β (Aβ)-related biomarkers (i.e. upstream biomarkers), such as Aβ levels detected through cerebrospinal fluid (CSF) measurements and positron emission tomography (PET). These changes precede structural alterations (i.e. downstream biomarkers) measured using magnetic resonance imaging (MRI) and computed tomography (CT). These pathological changes are followed by symptom manifestations (e.g. memory impairment). Therefore, changes in MEG oscillatory parameters potentially reflect pathological changes captured by midstream biomarkers. Previous studies have demonstrated the associations between MEG oscillatory characteristics and pathological biomarkers, such as Aβ deposition, cortical tau burden, glucose metabolism, synaptic density, and brain mass reduction (Fernández et al., 2003; Nakamura et al., 2018; Coomans et al., 2021). Although these studies have shown that MEG oscillatory parameters could be used to identify pathological changes, they were conducted in laboratory settings. It is important to investigate their relationship with other neuroimaging biomarkers sensitive to pathological conditions, currently used in clinical practice, to enhance the validity and comprehensibility of MEG oscillatory parameters as clinical examination tools for cognitive impairment.

This study examined these relationships using eZIS parameters as clinically validated pathological biomarkers. The eZIS parameters are neuroimaging biomarkers computed from SPECT measurements and are commonly used in clinical practice to assess individuals with cognitive decline (Matsuda et al., 2007; Imabayashi et al., 2017). The eZIS parameters consist of three values, namely *severity*, *extent*, and *ratio*, reflecting the probability of AD. This study revealed the following two main findings: (a) RPt positively correlated with *severity*, predominantly in the right hemisphere (Figures 1, 3A), and (b) IAF negatively correlated with *severity* in the caudal region (Figure 3B), indicating that the slower shift of the alpha peak corresponds to an increased likelihood of AD pathology.

The positive correlation between RPt and the eZIS parameter related to AD (*severity*) was found mainly in the right hemisphere (Figure 3A), which was replicated using the dataset from individuals with AD continuum (MCI + AD) (Supplementary Figure S4A; Supplementary Table S4A). The *severity* provides quantification of hypoperfusion in specific VOIs around the bilateral PCG, PC, and parietal lobe, the enhancement of which is linked to an increased pathological probability of AD (Matsuda et al., 2007). Its positive correlation with RPt suggests that augmentation of theta power corresponds to hypoperfusion in the VOIs, indicating a high probability of AD. This finding corroborates the observation of a previous EEG study of patients with AD, which demonstrated a close relationship between rCBF and quantitative EEG parameters in the theta band (Passero et al., 1995). Furthermore, a previous study demonstrated a negative correlation between RP in the 2–6 Hz range (spanning from delta to theta band) and global rCBF among both patients with AD and individuals with healthy ageing, particularly in the right hemisphere (Rodriguez et al., 1998). In a separate study, it was found that RP in the 2.0–5.5 Hz range (delta to theta band) exhibited a negative correlation with rCBF in the parietal and right hippocampal regions in patients with probable AD (Rodriguez et al., 1999). Additionally, a more recent study on patients with MCI demonstrated that a group with a low risk of conversion to AD showed a negative correlation between RPt and rCBF in the hippocampal complex (Moretti et al., 2013). Notably, enhancement of low-frequency oscillatory activity, including the RPt, is a known MEG oscillatory characteristic in patients with cognitive decline (Poza et al., 2007; Fernández et al., 2013; López et al., 2014). Consistently, the present study also demonstrated that global RPt negatively correlated with NPAs (Figures 1, 4, 5), suggesting that RPt is sensitive to both pathological and symptomatic changes accompanied by cognitive impairment. The pathological background of RPt has been studied using CSF biomarkers. For example, a previous study showed that RPt in the resting-state EEG data was correlated with both NPAs and total tau (T-tau) levels in CSF (Musaeus et al., 2018), while another EEG study demonstrated a correlation between CSF Aβ-42 concentration and the current source density over the right temporal area in the theta band (Hata et al., 2016). Moreover, EEG studies revealed that the combined ratio of the phosphorylated tau and Aβ42 (Aβ-42/p-tau ratio) positively correlated with theta power in the right posterior electrodes (Stomrud et al., 2010; Kramberger et al., 2013). A recent EEG study also reported increased resting-state delta and theta rhythms among patients with MCI with positive CSF biomarkers of Aβ-42/p-tau ratio (Jovicich et al., 2019). The relationship between cortical tau burden and the slowing of oscillatory activity in the occipital region was also found in a combined study of MEG and [^18^F] flortaucipir PET (Coomans et al., 2021). High CSF T-tau levels have also been demonstrated to be correlated with decreased rCBF in the right superior posterior medial frontal lobe (Stomrud et al., 2012), and it has been shown that the presence of Aβ deposition influences longitudinal changes in rCBF in humans (Sojkova et al., 2008) and animal models (Maier et al., 2014). Additionally, the eZIS parameter is sensitive to Aβ deposition (Takemaru et al., 2017). These findings suggest that the increase in RPt in the right hemisphere reflects the presence of CSF biomarkers, including Aβ deposition and T-tau levels. Furthermore, frontal and occipital theta power correlates with hippocampal atrophy (Grunwald et al., 2007; Nakamura et al., 2018), and global theta power correlates with memory function (Klimesch, 1999). Therefore, RPt may not only serve as the sole reflector of pathological changes but also reflect the symptomatic conditions of cognitive impairment. Notably, the right hemisphere plays an important role in the relationship between CSF biomarkers, hypoperfusion, and oscillatory changes in the RPt (Stomrud et al., 2010, 2012; Kramberger et al., 2013; Hata et al., 2016). We previously demonstrated that blood velocity was correlated with MF and IAF in the right common carotid artery (CCA) but not in the left CCA (Matsumoto et al., 2021). This finding suggests a hemispheric asymmetry in the neuropathological mechanisms underlying AD.

Our second finding [i.e. negative correlations between IAF and *severity* (Figure 3B)] indicates that changes in the alpha peak, characterised by slow shifts, are associated with hypoperfusion in the VOIs and increased pathological probability of AD. This result was also confirmed using the dataset from individuals with AD continuum (MCI + AD) (Supplementary Figure S4B; Supplementary Table S4B). This finding is consistent with that of a previous EEG study on patients with AD, which showed a significant positive correlation between the rCBF in the temporoparietal region of interest and the peak frequencies in the temporal and central electrodes (Kwa et al., 1993). Another study on patients with mild to moderate AD demonstrated that the MF, computed with a frequency range of 2–32 Hz, was positively correlated with rCBF in the parietal lobule, including the PC, superior parietal lobule, and postcentral gyrus (Rodriguez et al., 2004). The definition of the MF in the previous study (i.e. a frequency range of 2–32 Hz) (Rodriguez et al., 2004) was similar to that of the IAF in this study (i.e. a frequency range of 4–15 Hz), which accounted for the effects of a slow shift in the dominant frequency (i.e. alpha peak). This similarity indicates that the findings of the abovementioned study are suggestive of a potential association between slow shift of the alpha peak and hypoperfusion in the parietal lobule, including the PC, superior parietal lobule, and postcentral gyrus (Rodriguez et al., 2004). Notably, the relevance of alpha band power to the changes in cholinergic neurotransmission, one of the AD pathologies, has been reported previously. For example, the volumes of cholinergic cell clusters corresponding to the medial septum, vertical and horizontal limbs of the diagonal band, and posterior nucleus basalis of Meynert positively correlated with pre-alpha (i.e. a frequency range of 5.5–8hz) power in patients with MCI (Rea et al., 2021). Furthermore, an animal study showed that alpha power was decreased by experimental damage to this cholinergic pathway (Holschneider et al., 1998). A 1-year follow-up study demonstrated that a group of patients with mild AD who responded to pharmacological treatment with donepezil (i.e. acetylcholinesterase inhibitors) had a lesser magnitude reduction of occipital and temporal alpha sources than their non-responder counterparts (Babiloni et al., 2006b). The association between cholinergic deficits and EEG data was also examined using scopolamine, a non-selective muscarine receptor antagonist that blocks the stimulation of post-synaptic receptors. After scopolamine administration, decreases in EEG alpha power were observed in patients with AD and healthy controls (for reviews, see Ebert and Kirch, 1998; Jeong, 2004). Similarly, another study demonstrated a decrease in alpha power in the occipitotemporal regions in patients with AD (Vecchio et al., 2011), which could be an electrophysiological fingerprint of AD-specific pathological changes in cholinergicneurotransmission.

### 4.2 Symptomatic relevance of MEG oscillatory characteristics and eZIS parameters

This study demonstrated that MEG oscillatory parameters effectively capture the pathological changes represented by eZIS parameters (Figures 1, 3). Importantly, we found that MEG oscillatory parameters were not merely reflectors of the pathological conditions of patients. To explore their symptomatic significance, we evaluated the symptomatic relevance of MEG oscillatory and eZIS parameters with NPAs. We demonstrated that MEG oscillatory parameters correlated with both NPAs and eZIS parameters. However, direct correlations between the NPAs and eZIS parameters were sparse (Figure 2).

This study showed that global slowing and loss of diversity in neural oscillatory components were correlated with MMSE and FAB scores. Although differences in MEG oscillatory parameters between healthy ageing individuals and patients have been widely studied (Engels et al., 2017; López-Sanz et al., 2018; Mandal et al., 2018; Maestú et al., 2019), data on their links to NPAs are limited. A previous study investigated the correlations between RPs and various NPAs comprehensively (López et al., 2014), which reported that MMSE was correlated negatively with RPd and RPt and positively with RP in alpha band (8–12 Hz) and RPb. Additionally, it revealed that performance of the trail making test, an NPA for evaluating the executive functions, was correlated negatively with RPd and RPt but positively with RP in alpha band (8–12 Hz) and RPb (López et al., 2014). These results largely corroborated our findings, with the exception of the alpha band. Regarding the alpha band, we subdivided the range into alpha1 (7–9 Hz), alpha2 (9–11 Hz), and alpha3 (11–13 Hz), following the approach in a previous study (Wu and Liu, 1995). This subdivision was motivated by the expectation that their correlational behaviours with the level of cognitive impairments would be opposite before and after the alpha peak (Poza et al., 2007; Gómez et al., 2013; López et al., 2014); considering that the bands as a whole might lead to cancelation of these opposing effects. As expected, the behaviours of the lower alpha (alpha1) and higher alpha (alpha3) were distinctive; the former showed negative correlations, while the latter showed positive correlations with the NPAs (Figures 1, 4B,C, 5B,C; Supplementary Tables S7, S10B,C, S13B,C). In contrast, the middle alpha (alpha2) around the peak of the alpha activity showed no correlations with any of the parameters. The previous study (López et al., 2014) used a whole alpha band (8–12 Hz) in the correlational analysis but used subdivided alpha bands in the group level comparison, indicating that a-md-MCI group, who showed impairments in various cognitive domains, showed smaller occipital RPa2 and right frontotemporal RP in 10–12 Hz than for a-sd-MCI group, who exhibited an isolated memory impairment. This difference implied that the cognitive functions, except those in the memory domain, were responsive to the RPa2 and RP at 10–12 Hz. These results were also largely supported by our results, which showed that RPa2 demonstrated positive correlation coefficients with NPAs (Figure 1), although they were not statistically significant, and RPa3, which corresponds to the RP in 10–12 Hz in the previous study, exhibited positive correlations to the NPAs (Figures 1, 4C, 5C; Supplementary Tables S7, S10C, S13C). Other MEG studies have also reported a positive correlation between global MF and MMSE scores (Fernández et al., 2006) and a negative correlation between delta current densities and cognitive status (Fernández et al., 2013). Additionally, another MEG study parameterised the overall slowing of PSDn by calculating the ratio between RPs in faster and slower bands (e.g. RP in the alpha band/RP in the theta band) and showed that the slowing parameters were correlated with the MMSE score as well as with the individual RPs (i.e. RPd, RPt, and RPb) (Poza et al., 2008a). Notably, the ‘ratio’ parameters of the oscillatory powers should be interpreted with caution, particularly when the study did not clarify the cause of the changes, which may be attributed to the increase/decrease of paired oscillatory powers, but there are alternative accounts; for example, the ratio is heavily biased by the presence of an aperiodic component (see section 4.3). A recent study by our group showed that global MEG spectral parameters positively correlated with MMSE/FAB scores (Hoshi et al., 2022). The relationship between oscillatory parameters measured using EEG and NPAs has been described previously. For example, previous studies have shown negative correlations between parieto-occipital delta sources and MMSE scores (Babiloni et al., 2006a; Lizio et al., 2016), positive correlations between RPs in the alpha and beta bands in frontal electrodes and MMSE scores (Torabinikjeh et al., 2022), and positive correlations between the prefrontal MF, IAF, alpha-to-theta ratio, and MMSE scores after adjusting for age and education level (Choi et al., 2019). Moreover, other studies examined the relationships between EEG oscillatory parameters and MMSE scores in patients with probable AD using a coefficient of determination (*R*^2^), which quantifies the amount of data variation explained by MMSE; these studies revealed that the changes in RPs in the theta, alpha, and beta bands and SSE corresponded to changes in MMSE scores (Garn et al., 2014, 2015; Coronel et al., 2017). These findings, in line with our findings, indicate that the slowing and loss of complexity/diversity in neural oscillatory signals are associated with cognitive symptoms.

We found no significant correlations between the eZIS parameters and NPAs. It is important to note that the eZIS parameters—*severity*, *extent,* and *ratio—*were originally designed to maximise discriminating performance between aMCI and healthya ageing (i.e. maximising the area under the receiver operator curve), where the NPAs were used as one of the selection criteria of patients but not as parameters in the SPECT data analysis. For maximising the discriminating performance, the VOIs for the three parameters were set at the PCG, PC, and parietal lobe (Matsuda et al., 2007). Previous studies have shown that the MMSE score correlates with rCBF in the left hippocampus (Ikeda et al., 2008); frontal, parietal, and medial temporal cortices (Ushijima et al., 2002); and parietal and temporal cortices (Kimura et al., 2012). Similarly, another study revealed that the FAB score was correlated with rCBF in the left callosomarginal and precentral regions (Yoshida et al., 2009). However, it is worth noting that the VOIs used for computing the eZIS parameters (i.e. the PCG and PC) did not overlap with the regions where previous studies found correlations between rCBFs and MMSE or FAB scores. This finding indicates that the eZIS parameters exclusively capture hypoperfusion accompanied by pathological changes but do not reflect functional changes associated with symptoms of cognitive impairment. This notion is supported by a previous study that demonstrated how treatment with donepezil, a cholinesterase inhibitor used to effectively treat AD by enhancing cholinergic neurotransmission (Birks and Harvey, 2018), led to changes in rCBF in the PCC, along with changes in MMSE and AD assessment scale-cognitive scale scores (Iizuka and Kameyama, 2017). The eZIS parameters capture hypoperfusion in PCC, reflecting pathological changes in AD responsive to changes in cholinergic neurotransmission. The independence of eZIS parameters from symptomatic measurements was also exemplified by another study (Tokumitsu et al., 2021). The study revealed that the discrimination performance of patients with MCI and early AD was improved by subjecting MMSE and eZIS parameter (*extent*) in the logistic regression model, compared to the simple model including MMSE alone, indicating that the eZIS parameter contained non-overlapping and additive information to MMSE. The pathological changes do not always represent dementia symptoms (Snowdon, 1997; Shiroky et al., 2007). The three eZIS parameters may not be correlated with NPAs as they represent excerpts of rich information in rCBF, which were designed to capture the pathological changes.

### 4.3 Limitations

This study has four limitations. First, the raw rCBF data (i.e. whole brain data) were not used in the analysis for two reasons: (1) this study focused on clinically validated pathological biomarkers (i.e. eZIS parameters), and (2) the raw rCBF data were not available in the clinical records of the hospital. Therefore, the present results, such as the sparse correlations between eZIS parameters and NPAs, cannot be generalised to rCBF data. The hypoperfusion severity in regions other than the VOIs used for eZIS might capture symptomatic changes, but this aspect remains to be addressed. Second, for examining correlational relationships, the number of datasets was limited (*N* = 64). However, we did not calculate optimal sample size for two reasons: (1) this was a retrospective study, and the number of samples could not be modified following the power calculations, and (2) estimating sample sizes requires expected correlation coefficient sizes, which we could not speculate on due to the absence of previous studies examining the relationships between MEG and eZIS parameters. We anticipate future prospective studies with larger sample sizes. Third, this study did not account for the presence of an aperiodic component in the PSDn. The neural power spectra can be decomposed into a periodic component, reflecting true oscillatory activity, and an aperiodic component, which is 1/f-like activity modeled using a Lorentzian function (Donoghue et al., 2020). Similar to classical studies, this study assumed that changes in the neural power spectra reflected the changes in oscillatory activities associated with cognitive impairment. However, recent studies have demonstrated the contribution of aperiodic component as a predictor of cognitive impairment, which found that the aperiodic components differed between AD vs. FTD (Wang et al., 2023) and DLB/PD vs. MCI/control (Rosenblum et al., 2023), while other studies showed no differences in the aperiodic components among healthy controls, patients with MCI, and patients with AD (Azami et al., 2023; Kopčanová et al., 2023). In examining the correlations between RPs and NPAs, this study demonstrated that the correlational directions were flipped between low- (from RPd to RPa1) and high-frequency bands (from RPa3 to RPhg), with a border transition at RPa2 (Figures 1, 4, 5). This implies the contributions of the aperiodic component, which shifts rotationally with a fulcrum around the peak frequency (i.e. alpha band). Detailed analysis of our dataset, which includes comparing aperiodic and periodic components, will contribute to the unified understanding of the contradictory findings in the previous studies. Notably, even if the correlations were accounted by aperiodic components without contributions of oscillatory components, it would not undermine the clinical utility of the MEG oscillatory parameters. This is because the presence of aperiodic contaminations would not change the relationships between MEG oscillatory parameters and the symptomatic/pathological information. The discriminations between aperiodic and periodic components would be influential only when we discuss the neural mechanisms behind the correlations and interpretation of the results. Fourth, the MEG oscillatory parameters were used ‘as is’ without refinement or modification to examine their hindered relationships with the eZIS parameters and NPAs. Fine-tuned parameters may reveal associations not observed with the current settings. We did not revise the MEG oscillatory parameters because they have been used repeatedly in previous studies by us (Haraguchi et al., 2021; Matsumoto et al., 2021; Hoshi et al., 2022; Hirata et al., 2024) and others (Fernández et al., 2006; Poza et al., 2007, 2008a,b; Gómez et al., 2013), and we aimed to examine THEIR relationships to eZIS parameters, but not those of any other/new parameters. For example, the MEG oscillatory parameters can be adjusted individually; the International Federation of Clinical Neurophysiology (IFCN)–EEG research workgroup (Babiloni et al., 2020) encouraged adjusting the frequency bands individually for minimising the biases on the statistical tests. However, this requires modification of the existing MEG oscillatory parameters, which is not addressed in this study. The individual adjustment of the frequency bands would be crucial if the study was addressing the pathological mechanisms underlying the oscillatory changes in electrophysiological signals. The parameters should be modified accordingly if future studies address this research topic.

### 4.4 Conclusion

This study demonstrated that MEG oscillatory parameters correlated with both eZIS parameters and NPAs. These associations between MEG oscillatory and eZIS parameters (clinically validated pathological biomarkers) support the clinical validity of MEG oscillatory parameters. Our results suggest that theta power augmentation and slower shift of the alpha peak could serve as potential fingerprints of AD pathology, and the global slowing and loss of diversity in neural oscillatory components represent symptomatic changes. Moreover, eZIS parameters did not show correlations with NPAs. These findings demonstrate the potential of MEG data to enhance the clinical examination of patients with cognitive impairments, providing deeper insights into their clinical status. The electrophysiological (MEG) examination uniquely shows the symptomatic status of patients (i.e. NPAs), while pathological (eZIS) examination provides clinically validated information about the underlying cause of the cognitive impairments (i.e. AD). Their combined use in the clinical practice would improve the overall care of patients with cognitive impairments.

## Data availability statement

The datasets presented in this study can be found in online repositories. The names of the repository/repositories and accession number(s) can be found at: Hoshi, Hideyuki (2023), ‘MEG x SPECT study,’ Mendeley Data, V1, doi: 10.17632/h3bhhtxhjc.1.

## Ethics statement

The studies involving humans were approved by Ethics Committee of Kumagaya General Hospital. The studies were conducted in accordance with the local legislation and institutional requirements. Written informed consent for participation in this study was provided by the participants’ legal guardians/next of kin.

## Author contributions

HH: Conceptualisation, Data curation, Formal analysis, Investigation, Methodology, Software, Visualisation, Writing – original draft, Writing – review & editing. YH: Investigation, Supervision, Writing – review & editing. KF: Data curation, Investigation, Writing – review & editing. MK: Investigation, Writing – review & editing. YS: Conceptualisation, Data curation, Funding acquisition, Investigation, Project administration, Supervision, Writing – original draft, Writing – review & editing.

## Footnotes

^1^https://www.fil.ion.ucl.ac.uk/spm/

^2^http://neuroimage.usc.edu/brainstorm
