# Supplementary material for: Oscillatory characteristics of resting-state magnetoencephalography reflect pathological and symptomatic conditions of cognitive impairment
Source: Front Aging Neurosci. 2024 Jan 30;16:1273738. doi: 10.3389/fnagi.2024.1273738 (PMC10861731; doi:10.3389/fnagi.2024.1273738)
Supplement: Supplementary file 1 [file Data_Sheet_1.docx]

Supplementary Material

Oscillatory Characteristics of Resting-State Magnetoencephalography Reflect Pathological and Symptomatic Conditions of Cognitive Impairment

Hideyuki Hoshi^*^, Yoko Hirata, Keisuke Fukasawa, Momoko Kobayashi, Yoshihito Shigihara

*** Correspondence:**

Hideyuki Hoshi
heurekaesthem.avir@gmail.com

# Descriptive statistics details

**Table S1. Detailed profiles of individuals.**

|  | Healthy ageing  (N = 9, 5 females) | | | | MCI  (N = 15, 8 females) | | | | AD  (N = 30, 17 females) | | | | Other types of dementia  (N = 10, 5 females) | | | |
| --- | --- | --- | --- | --- | --- | --- | --- | --- | --- | --- | --- | --- | --- | --- | --- | --- |
|  | *M* | *SD* | MIN | MAX | *M* | *SD* | MIN | MAX | *M* | *SD* | MIN | MAX | *M* | *SD* | MIN | MAX |
| Age | 71.89 | 9.649 | 53 | 86 | 74.27 | 4.234 | 62 | 80 | 79.37 | 6.430 | 64 | 91 | 78.30 | 6.147 | 66 | 89 |
| MMSE | 29.44 | 0.527 | 29 | 30 | 27.40 | 0.986 | 26 | 29 | 22.37 | 3.987 | 11 | 28 | 19.90 | 7.310 | 9 | 30 |
| FAB | 15.00 | 1.225 | 13 | 17 | 13.93 | 1.792 | 11 | 18 | 10.57 | 2.932 | 4 | 16 | 10.33 | 3.162 | 7 | 17 |

AD, Alzheimer’s disease; MCI, mild cognitive impairment; *M*, mean; *SD*, standard deviation; MMSE, Mini-Mental State Examination; FAB, Frontal Assessment Battery

**
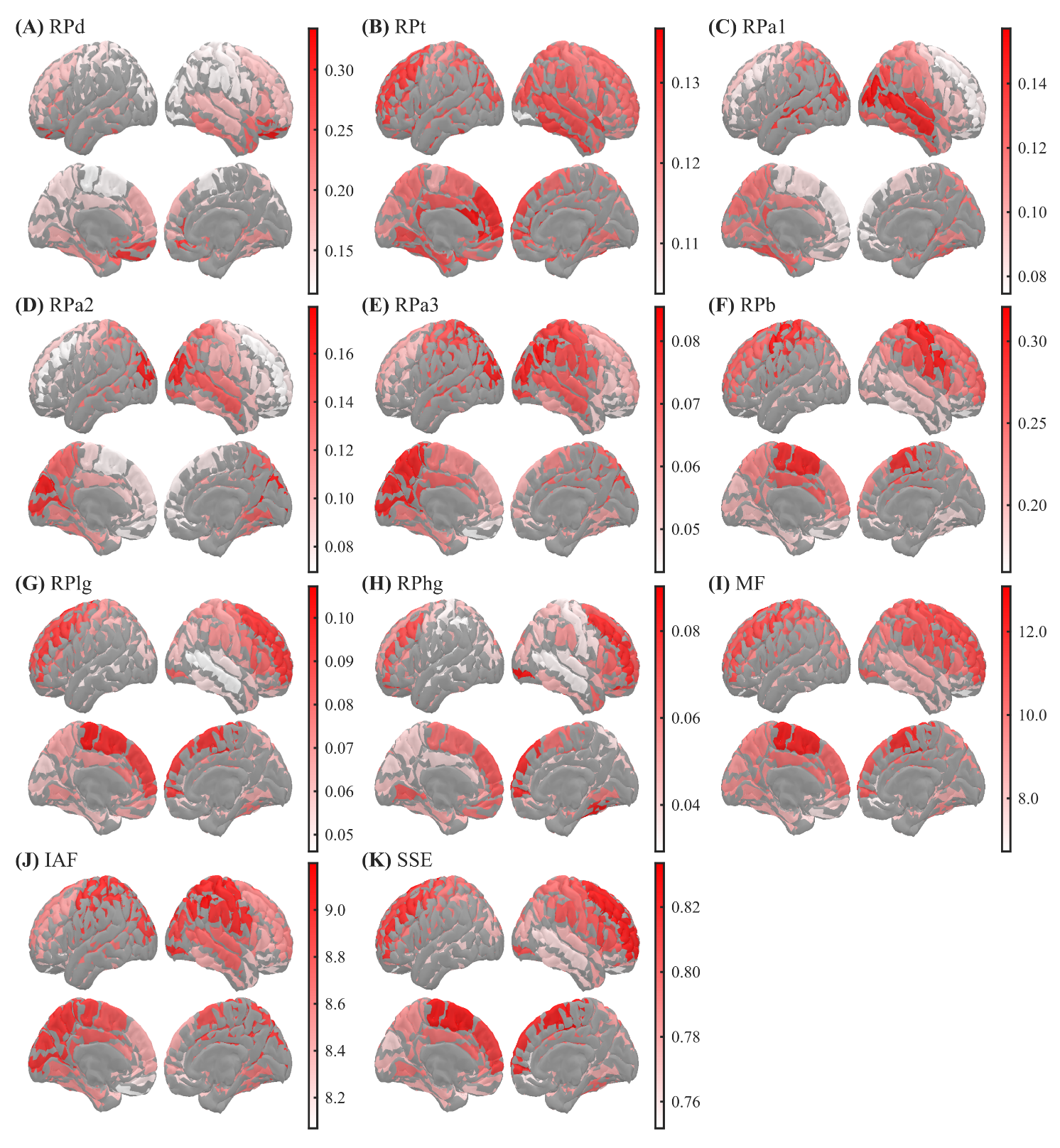
**

**Figure S1. Regional distribution of MEG oscillatory parameters.** MEG, magnetoencephalography; RPd, relative power in delta band; RPt, relative power in theta band; RPa1, relative power in alpha1 band; RPa2, relative power in alpha2 band; RPa3, relative power in alpha3 band; RPlg, relative power in low gamma band; RPhg, relative power in high gamma band; MF, mean frequency; IAF, individual alpha frequency; SSE, Shannon’s spectral entropy

# [AD+MCI] Results of global correlations between MEG oscillatory parameters, eZIS parameters, and NPAs


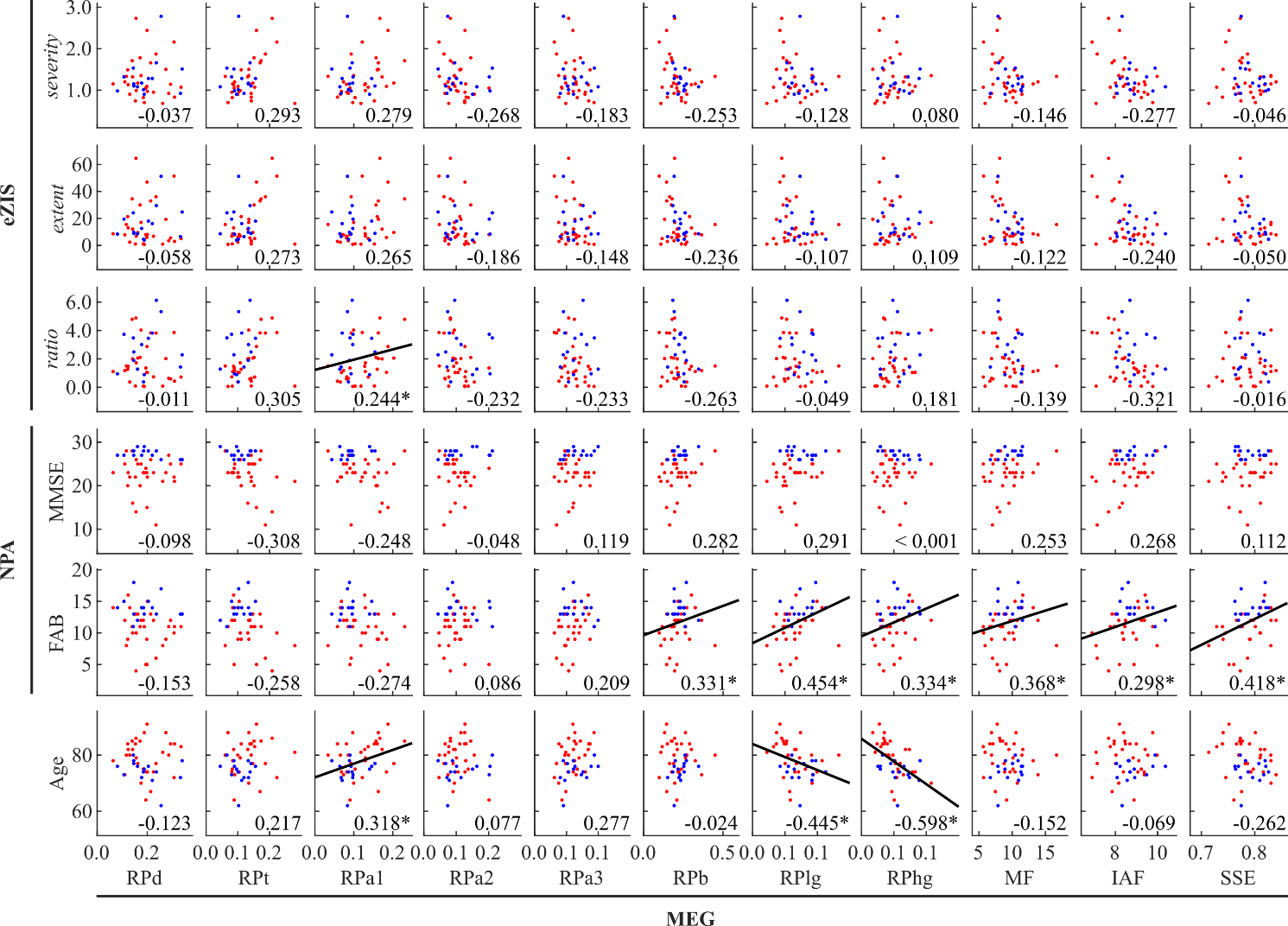


**Figure S2. [AD+MCI] Between-modality correlations between MEG oscillatory parameters averaged across anatomical regions, eZIS parameters, NPAs, and age.** The scatterplot is coloured differently for each diagnosis (blue: MCI and red: AD). Regression lines are added for significant correlations. The number displayed at the corner of each plot indicates Spearman’s coefficient (*rho*) averaged across bootstrap iterations, with an asterisk (*) indicating significant correlation. eZIS, easy Z-score imaging system; NPAs, neuropsychological assessments; MMSE, Mini-Mental State Examination; FAB, Frontal Assessment Battery; MEG, magnetoencephalography; RPd, relative power in delta band; RPt, relative power in theta band; RPa1, relative power in alpha1 band; RPa2, relative power in alpha2 band; RPa3, relative power in alpha3 band; RPb, relative power in beta band; RPlg, relative power in low gamma band; RPhg, relative power in high gamma band; MF, mean frequency; IAF, individual alpha frequency; SSE, Shannon’s spectral entropy; AD, Alzheimer’s disease; MCI, mild cognitive impairment

|  |  | MEG | | | | | | | | | | | |
| --- | --- | --- | --- | --- | --- | --- | --- | --- | --- | --- | --- | --- | --- |
|  |  | RPd | | | RPt | | | RPa1 | | | RPa2 | | |
|  |  | *rho* | *CI*(LL) | *CI*(UL) | *rho* | *CI*(LL) | *CI*(UL) | *rho* | *CI*(LL) | *CI*(UL) | *rho* | *CI*(LL) | *CI*(UL) |
| eZIS | *severity* | -0.037 | -0.323 | 0.247 | 0.293 | -0.040 | 0.586 | 0.279 | -0.001 | 0.543 | -0.268 | -0.529 | 0.058 |
|  | *extent* | -0.058 | -0.349 | 0.256 | 0.273 | -0.085 | 0.577 | 0.265 | -0.019 | 0.535 | -0.186 | -0.479 | 0.132 |
|  | *ratio* | -0.011 | -0.302 | 0.290 | 0.305 | -0.057 | 0.579 | 0.244* | 0.002 | 0.498 | -0.232 | -0.493 | 0.074 |
| NPA | MMSE | -0.098 | -0.371 | 0.176 | -0.308 | -0.575 | 0.023 | -0.248 | -0.532 | 0.079 | -0.048 | -0.363 | 0.283 |
|  | FAB | -0.153 | -0.428 | 0.145 | -0.258 | -0.528 | 0.053 | -0.274 | -0.523 | 0.017 | 0.086 | -0.208 | 0.366 |
|  | Age | -0.123 | -0.413 | 0.173 | 0.217 | -0.096 | 0.499 | 0.318* | 0.014 | 0.580 | 0.077 | -0.212 | 0.350 |

**Table S2. [AD+MCI] Between-modality correlations between MEG oscillatory parameters averaged across anatomical regions, eZIS parameters, NPAs, and age.**

*rho*, Spearman’s coefficient, averaged across bootstrap iterations; *CI*(LL), lower limit of 95% bootstrap confidence interval; *CI*(UL), upper limit of 95% bootstrap confidence interval; eZIS, easy Z-score imaging system; NPAs, neuropsychological assessments; MMSE, Mini-Mental State Examination; FAB, Frontal Assessment Battery; MEG, magnetoencephalography; RPd, relative power in delta band; RPt, relative power in theta band; RPa1, relative power in alpha1 band; RPa2, relative power in alpha2 band; AD, Alzheimer’s disease; MCI, mild cognitive impairment

**Table S2 (cont.). [AD+MCI] Between-modality correlations between MEG oscillatory parameters averaged across anatomical regions, eZIS parameters, NPAs, and age.**

|  |  | MEG | | | | | | | | | | | |
| --- | --- | --- | --- | --- | --- | --- | --- | --- | --- | --- | --- | --- | --- |
|  |  | RPa3 | | | RPb | | | RPlg | | | RPhg | | |
|  |  | *rho* | *CI*(LL) | *CI*(UL) | *rho* | *CI*(LL) | *CI*(UL) | *rho* | *CI*(LL) | *CI*(UL) | *rho* | *CI*(LL) | *CI*(UL) |
| eZIS | *severity* | -0.183 | -0.474 | 0.119 | -0.253 | -0.531 | 0.084 | -0.128 | -0.410 | 0.193 | 0.080 | -0.226 | 0.378 |
|  | *extent* | -0.148 | -0.453 | 0.167 | -0.236 | -0.525 | 0.095 | -0.107 | -0.404 | 0.233 | 0.109 | -0.189 | 0.414 |
|  | *ratio* | -0.233 | -0.515 | 0.074 | -0.263 | -0.496 | 0.053 | -0.049 | -0.339 | 0.279 | 0.181 | -0.095 | 0.470 |
| NPA | MMSE | 0.119 | -0.194 | 0.413 | 0.282 | -0.011 | 0.534 | 0.291 | -0.018 | 0.546 | < 0.001 | -0.313 | 0.302 |
|  | FAB | 0.209 | -0.071 | 0.464 | 0.332* | 0.015 | 0.572 | 0.454* | 0.134 | 0.682 | 0.334* | 0.053 | 0.580 |
|  | Age | 0.277 | -0.011 | 0.532 | -0.024 | -0.333 | 0.308 | -0.445* | -0.659 | -0.164 | -0.598* | -0.768 | -0.391 |

*rho*, Spearman’s coefficient, averaged across bootstrap iterations; *CI*(LL), lower limit of 95% bootstrap confidence interval; *CI*(UL), upper limit of 95% bootstrap confidence interval; eZIS, easy Z-score imaging system; NPAs, neuropsychological assessments; MMSE, Mini-Mental State Examination; FAB, Frontal Assessment Battery; MEG, magnetoencephalography; RPa3, relative power in alpha3 band; RPlg, relative power in low gamma band; RPhg, relative power in high gamma band; AD, Alzheimer’s disease; MCI, mild cognitive impairment

**Table S2 (cont.). [AD+MCI] Between-modality correlations between MEG oscillatory parameters averaged across anatomical regions, eZIS parameters, NPAs, and age.**

|  |  | MEG | | | | | | | | |
| --- | --- | --- | --- | --- | --- | --- | --- | --- | --- | --- |
|  |  | MF | | | IAF | | | SSE | | |
|  |  | *rho* | *CI*(LL) | *CI*(UL) | *rho* | *CI*(LL) | *CI*(UL) | *rho* | *CI*(LL) | *CI*(UL) |
| eZIS | *severity* | -0.146 | -0.439 | 0.189 | -0.277 | -0.565 | 0.090 | -0.046 | -0.347 | 0.257 |
|  | *extent* | -0.122 | -0.431 | 0.216 | -0.240 | -0.543 | 0.096 | -0.050 | -0.344 | 0.267 |
|  | *ratio* | -0.139 | -0.425 | 0.183 | -0.321 | -0.586 | 0.035 | -0.016 | -0.306 | 0.299 |
| NPA | MMSE | 0.253 | -0.029 | 0.500 | 0.268 | -0.058 | 0.543 | 0.112 | -0.198 | 0.387 |
|  | FAB | 0.368* | 0.068 | 0.604 | 0.298* | 0.002 | 0.563 | 0.418* | 0.129 | 0.654 |
|  | Age | -0.152 | -0.420 | 0.135 | -0.069 | -0.345 | 0.239 | -0.262 | -0.524 | 0.039 |

*rho*, Spearman’s coefficient, averaged across bootstrap iterations; *CI*(LL), lower limit of 95% bootstrap confidence interval; *CI*(UL), upper limit of 95% bootstrap confidence interval; eZIS, easy Z-score imaging system; NPAs, neuropsychological assessments; MMSE, Mini-Mental State Examination; FAB, Frontal Assessment Battery; MEG, magnetoencephalography; MF, mean frequency; IAF, individual alpha frequency; SSE, Shannon’s spectral entropy; AD, Alzheimer’s disease; MCI, mild cognitive impairment


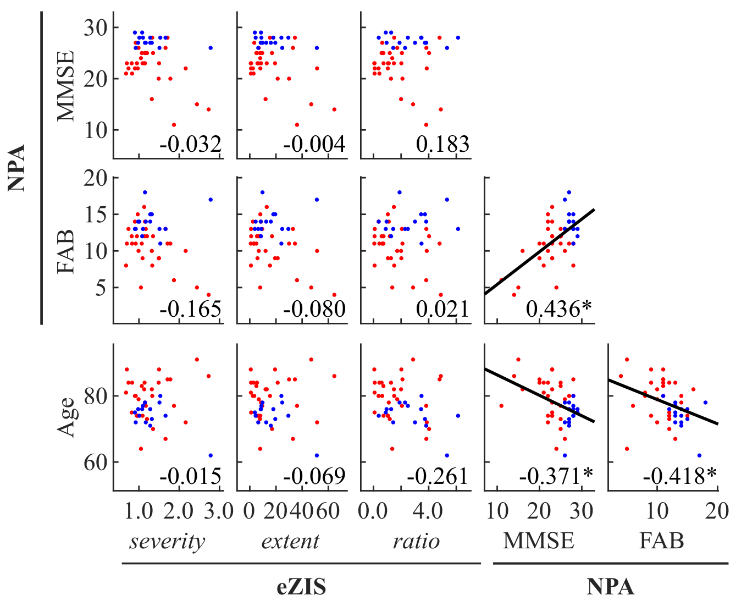


**Figure S3. [AD+MCI] Within- and between-modality correlations** **among eZIS parameters, NPAs, and age.** Regression lines are added for significant correlations. The scatterplot is coloured differently for each diagnosis (blue: MCI and red: AD). The number displayed at the corner of each plot indicate Spearman’s coefficient (*rho*) averaged across bootstrap iterations, with an asterisk (*) indicating significant correlation. eZIS, easy Z-score imaging system; NPAs, neuropsychological assessments; MMSE, Mini-Mental State Examination; FAB, Frontal Assessment Battery; AD, Alzheimer’s disease; MCI, mild cognitive impairment

|  |  | eZIS | | | | | | | | |
| --- | --- | --- | --- | --- | --- | --- | --- | --- | --- | --- |
|  |  | *severity* | | | *extent* | | | *ratio* | | |
|  |  | *rho* | *CI*(LL) | *CI*(UL) | *rho* | *CI*(LL) | *CI*(UL) | *rho* | *CI*(LL) | *CI*(UL) |
| NPA | MMSE | -0.032 | -0.373 | 0.321 | -0.004 | -0.347 | 0.326 | 0.183 | -0.142 | 0.479 |
|  | FAB | -0.165 | -0.453 | 0.167 | -0.080 | -0.390 | 0.251 | 0.021 | -0.275 | 0.308 |
|  | Age | -0.015 | -0.360 | 0.327 | -0.069 | -0.403 | 0.301 | -0.261 | -0.561 | 0.082 |

**Table S3. [AD+MCI] Within- and between-modality correlations among eZIS parameters, NPAs, and age.**

*rho*, Spearman’s coefficient, averaged across bootstrap iterations; *CI*(LL), lower limit of 95% bootstrap confidence interval; *CI*(UL), upper limit of 95% bootstrap confidence interval; eZIS, easy Z-score imaging system; NPAs, neuropsychological assessments; MMSE, Mini-Mental State Examination; FAB, Frontal Assessment Battery; AD, Alzheimer’s disease; MCI, mild cognitive impairment

**Table S3 (cont.). [AD+MCI] Within- and between-modality correlations among eZIS parameters, NPAs, and age.**

|  |  | NPA | | | | | |
| --- | --- | --- | --- | --- | --- | --- | --- |
|  |  | MMSE | | | FAB | | |
|  |  | *rho* | *CI*(LL) | *CI*(UL) | *rho* | *CI*(LL) | *CI*(UL) |
| NPA | FAB | 0.436* | 0.131 | 0.671 |  |  |  |
|  | Age | -0.371* | -0.598 | -0.105 | -0.418* | -0.647 | -0.099 |

*rho*, Spearman’s coefficient, averaged across bootstrap iterations; *CI*(LL), lower limit of 95% bootstrap confidence interval; *CI*(UL), upper limit of 95% bootstrap confidence interval; eZIS, easy Z-score imaging system; NPAs, neuropsychological assessments; MMSE, Mini-Mental State Examination; FAB, Frontal Assessment Battery; AD, Alzheimer’s disease; MCI, mild cognitive impairment

# [AD+MCI] Results of regional correlations between MEG oscillatory and eZIS parameters

**
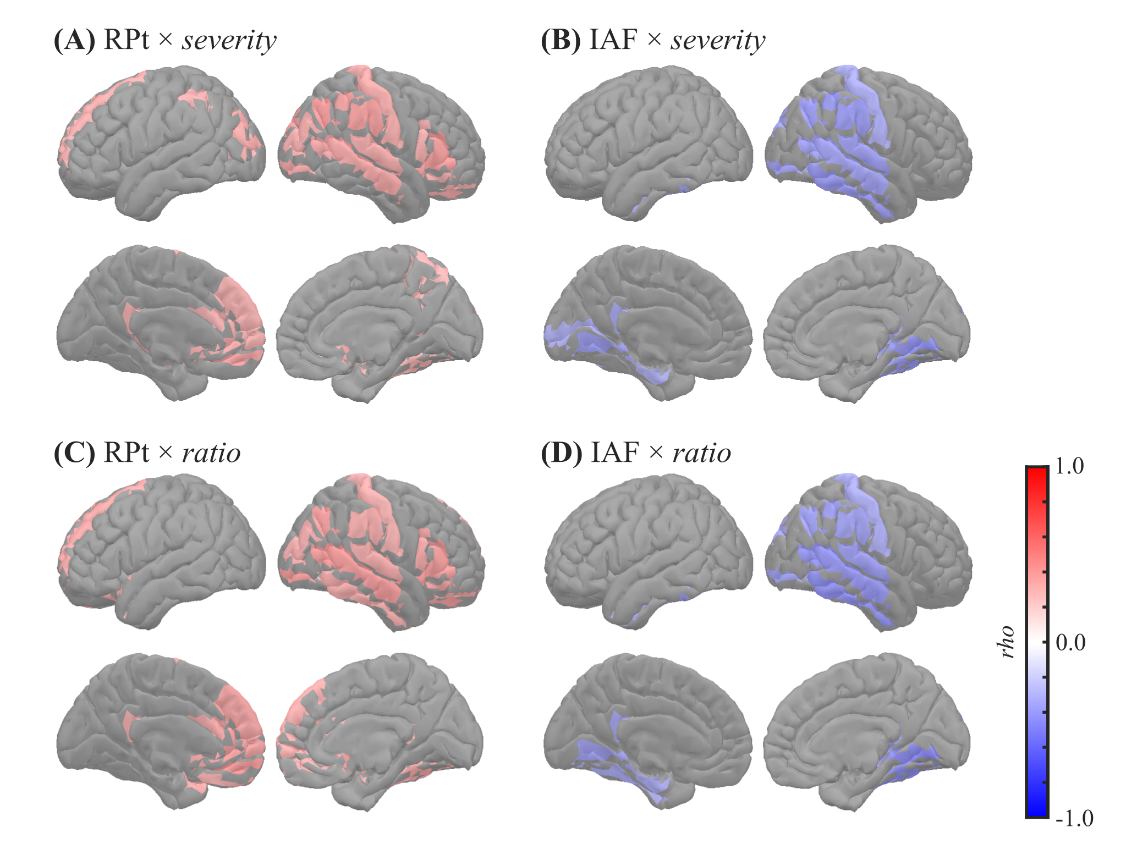
**

**Figure S4. [AD+MCI] Regional correlations between MEG oscillatory and eZIS parameters.** Cortical projection of significant clusters (*P* < 0.025), indicated by coloured regions with correlation coefficients between (A) RPt and *severity*, (B) IAF and *severity*, (C) RPt and *ratio*, and (D) IAF and *ratio*. *rho*, Spearman’s coefficient; MEG, magnetoencephalography; RPt, relative power in theta band; IAF, individual alpha frequency; eZIS, easy Z-score imaging system; AD, Alzheimer’s disease; MCI, mild cognitive impairment

**Table S4. [AD+MCI] Regions and statistical values included in clusters for correlations between MEG oscillatory and eZIS parameters**

|  |  |  | (A) RPt × *severity* | |  | (B) IAF × *severity* | |
| --- | --- | --- | --- | --- | --- | --- | --- |
| Region | LR |  | *rho* | *T* |  | *rho* | *T* |
| Amygdala | R |  | 0.329 | 2.281 |  |  |  |
| Angular gyrus | R |  | 0.398 | 2.848 |  | -0.385 | -2.735 |
| Anterior cingulate cortex-pregenual | L |  | 0.337 | 2.351 |  |  |  |
| Anterior cingulate cortex-subgenual | L |  | 0.334 | 2.320 |  |  |  |
|  | R |  | 0.324 | 2.248 |  |  |  |
| Anterior cingulate cortex-supracallosal | L |  | 0.302 | 2.079 |  |  |  |
| Anterior orbital gyrus | R |  | 0.297 | 2.039 |  |  |  |
| Calcarine fissure and surrounding cortex | L |  |  |  |  | -0.313 | -2.163 |
| Caudate nucleus | L |  | 0.293 | 2.012 |  |  |  |
| Crus I of the cerebellar hemisphere | L |  |  |  |  | -0.365 | -2.574 |
|  | R |  | 0.331 | 2.303 |  | -0.431 | -3.133 |
| Fusiform gyrus | R |  | 0.298 | 2.045 |  | -0.385 | -2.732 |
| Gyrus rectus | R |  | 0.319 | 2.209 |  |  |  |
| Heschl’s gyrus | R |  | 0.313 | 2.162 |  | -0.362 | -2.545 |
| Hippocampus | L |  | 0.356 | 2.498 |  |  |  |
|  | R |  |  |  |  | -0.330 | -2.293 |
| IFG pars orbitalis | R |  | 0.311 | 2.144 |  |  |  |
| Inferior frontal gyrus-opercular part | R |  | 0.307 | 2.118 |  |  |  |
| Inferior frontal gyrus-triangular part | R |  | 0.383 | 2.722 |  |  |  |
| Inferior occipital gyrus | L |  |  |  |  | -0.317 | -2.195 |
|  | R |  | 0.333 | 2.315 |  | -0.373 | -2.637 |
| Inferior parietal gyrus-excluding supramarginal and angular gyri | L |  | 0.305 | 2.101 |  |  |  |
|  | R |  | 0.357 | 2.509 |  | -0.391 | -2.785 |
| Inferior temporal gyrus | L |  |  |  |  | -0.326 | -2.264 |
|  | R |  |  |  |  | -0.400 | -2.862 |
| Insula | R |  | 0.368 | 2.595 |  |  |  |
| Lateral orbital gyrus | R |  | 0.337 | 2.349 |  |  |  |
| Lingual gyrus | L |  |  |  |  | -0.370 | -2.613 |
|  | R |  |  |  |  | -0.420 | -3.038 |
| Lobule IV-V of the cerebellar hemisphere | L |  |  |  |  | -0.347 | -2.423 |
|  | R |  |  |  |  | -0.293 | -2.011 |
| Lobule IV-V of vermis |  |  |  |  |  | -0.346 | -2.416 |
| Lobule VI of the cerebellar hemisphere | R |  | 0.352 | 2.468 |  | -0.429 | -3.113 |
| Medial orbital gyrus | R |  | 0.338 | 2.353 |  |  |  |
| Middle occipital gyrus | L |  | 0.324 | 2.242 |  |  |  |
|  | R |  | 0.313 | 2.158 |  |  |  |
| Middle temporal gyrus | R |  | 0.348 | 2.437 |  | -0.364 | -2.564 |
| Olfactory cortex | L |  | 0.349 | 2.442 |  |  |  |
|  | R |  | 0.371 | 2.618 |  |  |  |
| Parahippocampal gyrus | L |  |  |  |  | -0.299 | -2.055 |
| Postcentral gyrus | R |  | 0.327 | 2.270 |  | -0.317 | -2.194 |
| Posterior cingulate gyrus | L |  | 0.317 | 2.189 |  | -0.341 | -2.382 |
|  | R |  |  |  |  | -0.351 | -2.458 |
| Precuneus | R |  | 0.300 | 2.062 |  |  |  |
| Pulvinar medial | L |  | 0.365 | 2.569 |  | -0.367 | -2.586 |
| Superior frontal gyrus-dorsolateral | L |  | 0.315 | 2.178 |  |  |  |
| Superior frontal gyrus-medial | L |  | 0.310 | 2.138 |  |  |  |
| Superior frontal gyrus-medial orbital | L |  | 0.368 | 2.592 |  |  |  |
| Superior occipital gyrus | R |  | 0.369 | 2.603 |  | -0.351 | -2.462 |
| Superior temporal gyrus | R |  | 0.333 | 2.313 |  | -0.382 | -2.712 |
| SupraMarginal gyrus | R |  | 0.392 | 2.791 |  | -0.395 | -2.821 |
| Mean | |  | 0.335 | 2.334 |  | -0.361 | -2.547 |

**(A) RPt** × ***severity*, and (B) IAF** × ***severity*.** Empty cells indicate the regions are not included in the cluster. AD, Alzheimer’s disease; MCI, mild cognitive impairment; eZIS, easy Z-score imaging system; RPt, relative power in theta band; IAF, individual alpha frequency; *rho*, Spearman’s coefficient; *T*, *T*-statistic.

**Table S5. [AD+MCI] Regions and statistical values included in clusters for correlations between MEG oscillatory and eZIS parameters.**

|  |  |  | (A) RPt × *ratio* | |  | (B) IAF × *ratio* | |
| --- | --- | --- | --- | --- | --- | --- | --- |
| Region | LR |  | *rho* | *T* |  | *rho* | *T* |
| Amygdala | L |  |  |  |  | -0.312 | -2.155 |
|  | R |  | 0.341 | 2.379 |  |  |  |
| Angular gyrus | R |  | 0.378 | 2.679 |  | -0.371 | -2.617 |
| Anterior cingulate cortex-pregenual | L |  | 0.387 | 2.752 |  |  |  |
| Anterior cingulate cortex-subgenual | L |  | 0.342 | 2.389 |  |  |  |
|  | R |  | 0.362 | 2.545 |  |  |  |
| Anterior cingulate cortex-supracallosal | L |  | 0.341 | 2.381 |  |  |  |
|  | R |  | 0.301 | 2.072 |  |  |  |
| Anterior orbital gyrus | R |  | 0.344 | 2.405 |  |  |  |
| Caudate nucleus | L |  | 0.314 | 2.170 |  |  |  |
| Crus I of cerebellar hemisphere | L |  |  |  |  | -0.358 | -2.512 |
|  | R |  | 0.393 | 2.803 |  | -0.490 | -3.689 |
| Fusiform gyrus | L |  |  |  |  | -0.321 | -2.219 |
|  | R |  | 0.328 | 2.275 |  | -0.436 | -3.180 |
| Gyrus rectus | L |  | 0.314 | 2.172 |  |  |  |
|  | R |  | 0.345 | 2.411 |  |  |  |
| Heschl’s gyrus | R |  | 0.306 | 2.105 |  | -0.404 | -2.898 |
| Hippocampus | R |  |  |  |  | -0.364 | -2.559 |
| IFG pars orbitalis | R |  | 0.356 | 2.500 |  |  |  |
| Inferior frontal gyrus-opercular part | R |  | 0.339 | 2.360 |  |  |  |
| Inferior frontal gyrus-triangular part | R |  | 0.389 | 2.766 |  |  |  |
| Inferior occipital gyrus | L |  |  |  |  | -0.323 | -2.240 |
|  | R |  | 0.376 | 2.657 |  | -0.365 | -2.570 |
| Inferior parietal gyrus-excluding supramarginal and angular gyri | R |  |  |  |  | -0.371 | -2.622 |
| Inferior temporal gyrus | L |  |  |  |  | -0.343 | -2.397 |
|  | R |  | 0.318 | 2.199 |  | -0.427 | -3.093 |
| Insula | R |  | 0.394 | 2.810 |  |  |  |
| Lateral orbital gyrus | R |  | 0.367 | 2.584 |  |  |  |
| Lingual gyrus | L |  |  |  |  | -0.360 | -2.532 |
|  | R |  |  |  |  | -0.456 | -3.359 |
| Lobule IV-V of cerebellar hemisphere | L |  |  |  |  | -0.353 | -2.476 |
|  | R |  |  |  |  | -0.348 | -2.434 |
| Lobule IV-V of vermis |  |  | 0.301 | 2.068 |  | -0.372 | -2.628 |
| Lobule VI of cerebellar hemisphere | R |  | 0.377 | 2.670 |  | -0.452 | -3.320 |
| Medial orbital gyrus | R |  | 0.379 | 2.685 |  |  |  |
| Middle occipital gyrus | R |  | 0.310 | 2.134 |  |  |  |
| Middle temporal gyrus | R |  | 0.400 | 2.860 |  | -0.418 | -3.014 |
| Olfactory cortex | L |  | 0.351 | 2.454 |  |  |  |
|  | R |  | 0.398 | 2.844 |  |  |  |
| Parahippocampal gyrus | L |  |  |  |  | -0.315 | -2.174 |
| Postcentral gyrus | R |  | 0.304 | 2.090 |  | -0.316 | -2.181 |
| Posterior cingulate gyrus | L |  | 0.318 | 2.202 |  | -0.352 | -2.468 |
|  | R |  | 0.303 | 2.087 |  | -0.380 | -2.692 |
| Posterior orbital gyrus | L |  | 0.310 | 2.134 |  |  |  |
|  | R |  | 0.302 | 2.077 |  |  |  |
| Pulvinar medial | L |  | 0.342 | 2.386 |  | -0.374 | -2.646 |
| Superior frontal gyrus-dorsolateral | L |  | 0.340 | 2.372 |  |  |  |
| Superior frontal gyrus-medial | L |  | 0.362 | 2.546 |  |  |  |
|  | R |  | 0.330 | 2.293 |  |  |  |
| Superior frontal gyrus-medial orbital | L |  | 0.404 | 2.900 |  |  |  |
|  | R |  | 0.312 | 2.153 |  |  |  |
| Superior occipital gyrus | R |  |  |  |  | -0.332 | -2.309 |
| Superior temporal gyrus | R |  | 0.331 | 2.299 |  | -0.386 | -2.743 |
| SupraMarginal gyrus | R |  | 0.328 | 2.276 |  | -0.365 | -2.572 |
| Temporal pole: middle temporal gyrus | L |  | 0.307 | 2.118 |  | -0.341 | -2.376 |
| Temporal pole: superior temporal gyrus | L |  | 0.339 | 2.365 |  |  |  |
| Mean | |  | 0.344 | 2.405 |  | -0.373 | -2.644 |

**(A) RPt** × ***ratio*, and (B) IAF** × ***ratio*.** Empty cells indicate the regions are not included in the cluster. AD, Alzheimer’s disease; MCI, mild cognitive impairment; eZIS, easy Z-score imaging system; RPt, relative power in theta band; IAF, individual alpha frequency; *rho*, Spearman’s coefficient; *T*, *T*-statistic

# [AD+MCI] Results of regional correlations between MEG oscillatory parameters and NPAs


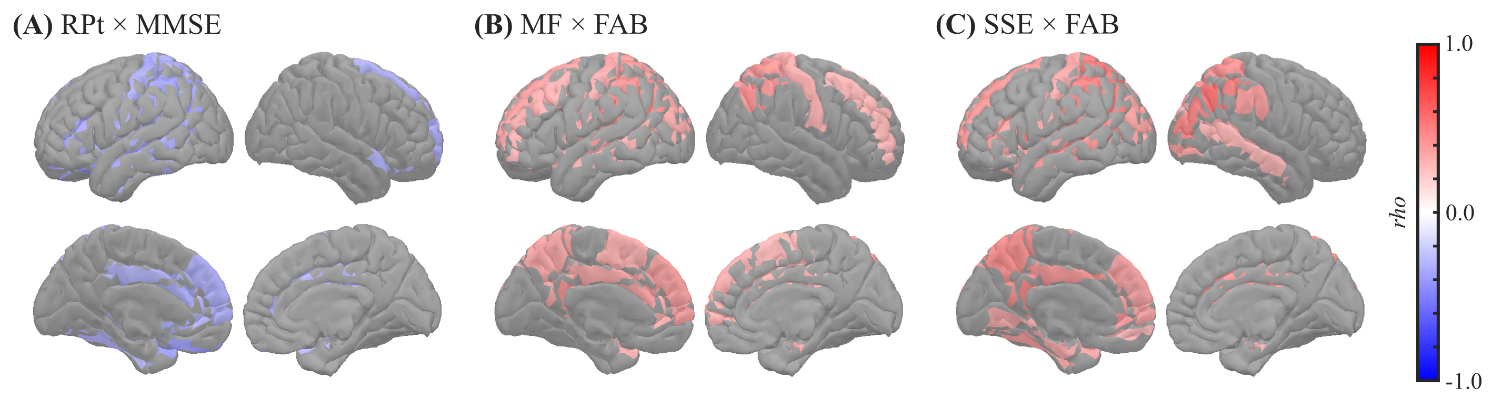


**Figure S5. [AD+MCI] Regional correlations between MEG oscillatory parameters and NPAs.** Cortical projection of significant clusters (*P* < 0.025), indicated by coloured regions with correlation coefficients between (A) RPt and MMSE, (B) MF and FAB, and (C) SSE and FAB. AD, Alzheimer’s disease; MCI, mild cognitive impairment; *rho*, Spearman’s coefficient; MEG, magnetoencephalography; RPt, relative power in theta band; MF, mean frequency; SSE, Shannon’s spectral entropy; NPAs, neuropsychological assessments; MMSE, Mini-Mental State Examination; FAB, Frontal Assessment Battery

**Table S6. [AD+MCI] Regions and statistical values included in clusters for correlations between MEG oscillatory parameters and NPA.**

|  |  |  | (A) RPt × MMSE | |  | (B) MF × FAB | |  | (C) SSE × FAB | |
| --- | --- | --- | --- | --- | --- | --- | --- | --- | --- | --- |
| Region | LR |  | *rho* | *T* |  | *rho* | *T* |  | *rho* | *T* |
| Amygdala | L |  |  |  |  | 0.329 | 2.284 |  | 0.355 | 2.494 |
| Angular gyrus | L |  | -0.396 | -2.826 |  | 0.466 | 3.457 |  | 0.551 | 4.329 |
|  | R |  |  |  |  | 0.457 | 3.367 |  | 0.585 | 4.729 |
| Anterior cingulate cortex-pregenual | L |  | -0.307 | -2.115 |  | 0.322 | 2.233 |  | 0.422 | 3.057 |
| Anterior cingulate cortex-supracallosal | L |  | -0.339 | -2.363 |  | 0.377 | 2.672 |  |  |  |
|  | R |  |  |  |  | 0.355 | 2.486 |  | 0.441 | 3.223 |
| Anterior orbital gyrus | R |  |  |  |  |  |  |  |  |  |
| Calcarine fissure and surrounding cortex | L |  |  |  |  |  |  |  |  |  |
|  | R |  |  |  |  |  |  |  |  |  |
| Crus I of the cerebellar hemisphere | L |  |  |  |  | 0.351 | 2.462 |  | 0.424 | 3.067 |
|  | R |  |  |  |  |  |  |  |  |  |
| Cuneus | L |  |  |  |  |  |  |  |  |  |
|  | R |  |  |  |  |  |  |  |  |  |
| Fusiform gyrus | L |  | -0.307 | -2.115 |  |  |  |  | 0.391 | 2.789 |
|  | R |  |  |  |  |  |  |  |  |  |
| Heschl’s gyrus | L |  | -0.420 | -3.039 |  | 0.392 | 2.790 |  |  |  |
|  | R |  |  |  |  |  |  |  |  |  |
| Hippocampus | R |  |  |  |  |  |  |  |  |  |
| IFG pars orbitalis | R |  |  |  |  |  |  |  |  |  |
| Inferior frontal gyrus-opercular part | L |  |  |  |  | 0.325 | 2.255 |  | 0.530 | 4.101 |
|  | R |  |  |  |  |  |  |  |  |  |
| Inferior frontal gyrus-triangular part | L |  | -0.336 | -2.336 |  |  |  |  | 0.360 | 2.529 |
|  | R |  |  |  |  |  |  |  |  |  |
| Inferior occipital gyrus | L |  |  |  |  | 0.325 | 2.252 |  | 0.420 | 3.038 |
| Inferior parietal gyrus-excluding supramarginal and angular gyri | L |  | -0.365 | -2.568 |  | 0.431 | 3.136 |  | 0.502 | 3.809 |
|  | R |  |  |  |  | 0.406 | 2.915 |  | 0.497 | 3.757 |
| Inferior temporal gyrus | R |  |  |  |  |  |  |  |  |  |
| Insula | L |  | -0.384 | -2.726 |  |  |  |  | 0.364 | 2.563 |
|  | R |  |  |  |  |  |  |  |  |  |
| Lateral orbital gyrus | L |  |  |  |  | 0.328 | 2.275 |  | 0.346 | 2.421 |
| Lingual gyrus | R |  |  |  |  |  |  |  |  |  |
| Lobule IV-V of the cerebellar hemisphere | R |  |  |  |  |  |  |  |  |  |
| Lobule IV-V of vermis |  |  |  |  |  |  |  |  |  |  |
| Lobule VI of cerebellar hemisphere | R |  |  |  |  |  |  |  |  |  |
| Medial orbital gyrus | R |  |  |  |  |  |  |  |  |  |
| Mediodorsal medial magnocellular | R |  | -0.321 | -2.223 |  | 0.327 | 2.265 |  |  |  |
| Middle cingulate & paracingulate gyri | L |  | -0.306 | -2.104 |  | 0.362 | 2.544 |  | 0.423 | 3.064 |
|  | R |  | -0.310 | -2.135 |  | 0.322 | 2.229 |  | 0.409 | 2.942 |
| Middle occipital gyrus | L |  |  |  |  | 0.344 | 2.401 |  | 0.388 | 2.762 |
| Middle temporal gyrus | L |  | -0.351 | -2.455 |  | 0.312 | 2.150 |  | 0.434 | 3.160 |
| Olfactory cortex | R |  |  |  |  |  |  |  |  |  |
| Paracentral lobule | L |  |  |  |  |  |  |  |  |  |
|  | R |  |  |  |  |  |  |  |  |  |
| Parahippocampal gyrus | R |  |  |  |  | 0.361 | 2.538 |  | 0.343 | 2.395 |
| Postcentral gyrus | L |  | -0.338 | -2.358 |  | 0.337 | 2.345 |  | 0.347 | 2.423 |
| Posterior cingulate gyrus | L |  |  |  |  | 0.440 | 3.214 |  | 0.494 | 3.730 |
|  | R |  |  |  |  |  |  |  |  |  |
| Posterior orbital gyrus | L |  | -0.443 | -3.241 |  |  |  |  | 0.335 | 2.331 |
| Precentral gyrus | L |  |  |  |  |  |  |  |  |  |
|  | R |  |  |  |  |  |  |  |  |  |
| Precuneus | L |  |  |  |  | 0.359 | 2.519 |  | 0.471 | 3.499 |
|  | R |  |  |  |  |  |  |  |  |  |
| Pulvinar medial | L |  |  |  |  |  |  |  |  |  |
| Rolandic operculum | R |  |  |  |  |  |  |  |  |  |
| Substantia nigra-pars compacta | R |  |  |  |  | 0.370 | 2.611 |  | 0.361 | 2.542 |
| Substantia nigra-pars reticulata | R |  |  |  |  | 0.344 | 2.405 |  | 0.330 | 2.293 |
| Superior frontal gyrus-dorsolateral | L |  |  |  |  | 0.375 | 2.652 |  | 0.416 | 3.000 |
| Superior frontal gyrus-medial | L |  | -0.327 | -2.271 |  | 0.383 | 2.720 |  | 0.383 | 2.717 |
| Superior occipital gyrus | L |  |  |  |  | 0.332 | 2.304 |  | 0.409 | 2.938 |
| Superior parietal gyrus | L |  | -0.329 | -2.288 |  | 0.395 | 2.818 |  | 0.529 | 4.089 |
|  | R |  |  |  |  | 0.409 | 2.938 |  | 0.441 | 3.218 |
| Superior temporal gyrus | L |  | -0.354 | -2.485 |  | 0.435 | 3.165 |  | 0.387 | 2.754 |
|  | R |  |  |  |  |  |  |  |  |  |
| SupraMarginal gyrus | L |  | -0.348 | -2.432 |  | 0.456 | 3.361 |  | 0.482 | 3.612 |
| Temporal pole: middle temporal gyrus | R |  |  |  |  |  |  |  |  |  |
| Temporal pole: superior temporal gyrus | L |  | -0.389 | -2.771 |  | 0.329 | 2.288 |  | 0.356 | 2.495 |
| Mean | |  | -0.351 | -2.466 |  | 0.370 | 2.627 |  | 0.422 | 3.087 |

**(A) RPt × MMSE, (B) MF × FAB, (C) SSE × FAB.** Empty cells indicate the regions are not included in the cluster. Regions where age showed potential confounding effects (*i.e.* larger variable importance in projection score in partial least square regression than FAB) were excluded from the list. AD, Alzheimer’s disease; MCI, mild cognitive impairment; RPt, relative power in theta band; MF, mean frequency; SSE, Shannon’s spectral entropy; MMSE, Mini-Mental State Examination; FAB, Frontal Assessment Battery; *rho*, Spearman’s coefficient; *T*, *T*-statistic

# Results of global correlations between MEG oscillatory parameters, eZIS parameters, and NPAs

**Table S7. Between-modality correlations between MEG oscillatory parameters averaged across anatomical regions, eZIS parameters, NPAs, and age.**

|  |  | MEG | | | | | | | | | | | |
| --- | --- | --- | --- | --- | --- | --- | --- | --- | --- | --- | --- | --- | --- |
|  |  | RPd | | | RPt | | | RPa1 | | | RPa2 | | |
|  |  | *rho* | *CI*(LL) | *CI*(UL) | *rho* | *CI*(LL) | *CI*(UL) | *rho* | *CI*(LL) | *CI*(UL) | *rho* | *CI*(LL) | *CI*(UL) |
| eZIS | *severity* | 0.016 | -0.240 | 0.256 | 0.270* | 0.009 | 0.527 | 0.189 | -0.062 | 0.434 | -0.219 | -0.434 | 0.023 |
|  | *extent* | -0.018 | -0.264 | 0.244 | 0.237 | -0.043 | 0.498 | 0.184 | -0.054 | 0.416 | -0.128 | -0.365 | 0.117 |
|  | *ratio* | -0.031 | -0.261 | 0.205 | 0.220 | -0.044 | 0.452 | 0.126 | -0.099 | 0.350 | -0.127 | -0.356 | 0.124 |
| NPA | MMSE | -0.203 | -0.411 | 0.034 | -0.420* | -0.608 | -0.174 | -0.350* | -0.540 | -0.117 | 0.143 | -0.114 | 0.377 |
|  | FAB | -0.214 | -0.428 | 0.026 | -0.353* | -0.580 | -0.082 | -0.290* | -0.485 | -0.070 | 0.219 | -0.037 | 0.443 |
|  | Age | -0.143 | -0.390 | 0.114 | 0.181 | -0.083 | 0.410 | 0.372* | 0.127 | 0.576 | -0.042 | -0.272 | 0.190 |

*rho*, Spearman’s coefficient, averaged across bootstrap iterations; *CI*(LL), lower limit of 95% bootstrap confidence interval; *CI*(UL), upper limit of 95% bootstrap confidence interval; eZIS, easy Z-score imaging system; NPAs, neuropsychological assessments; MMSE, Mini-Mental State Examination; FAB, Frontal Assessment Battery; MEG, magnetoencephalography; RPd, relative power in delta band; RPt, relative power in theta band; RPa1, relative power in alpha1 band; RPa2, relative power in alpha2 band

**Table S7 (cont.). Between-modality correlations between MEG oscillatory parameters averaged across anatomical regions, eZIS parameters, NPAs, and age.**

|  |  | MEG | | | | | | | | | | | |
| --- | --- | --- | --- | --- | --- | --- | --- | --- | --- | --- | --- | --- | --- |
|  |  | RPa3 | | | RPb | | | RPlg | | | RPhg | | |
|  |  | *rho* | *CI*(LL) | *CI*(UL) | *rho* | *CI*(LL) | *CI*(UL) | *rho* | *CI*(LL) | *CI*(UL) | *rho* | *CI*(LL) | *CI*(UL) |
| eZIS | *severity* | -0.151 | -0.393 | 0.110 | -0.246 | -0.492 | 0.015 | -0.053 | -0.318 | 0.201 | 0.078 | -0.193 | 0.332 |
|  | *extent* | -0.126 | -0.368 | 0.121 | -0.223 | -0.473 | 0.042 | -0.071 | -0.314 | 0.204 | 0.057 | -0.204 | 0.304 |
|  | *ratio* | -0.117 | -0.365 | 0.127 | -0.192 | -0.417 | 0.056 | 0.019 | -0.229 | 0.269 | 0.111 | -0.134 | 0.353 |
| NPA | MMSE | 0.365* | 0.139 | 0.562 | 0.430* | 0.214 | 0.618 | 0.322* | 0.092 | 0.519 | 0.016 | -0.236 | 0.263 |
|  | FAB | 0.377* | 0.154 | 0.572 | 0.390* | 0.120 | 0.602 | 0.431* | 0.190 | 0.625 | 0.206 | -0.030 | 0.431 |
|  | Age | 0.145 | -0.115 | 0.384 | 0.031 | -0.228 | 0.296 | -0.278* | -0.481 | -0.032 | -0.349* | -0.555 | -0.117 |

*rho*, Spearman’s coefficient, averaged across bootstrap iterations; *CI*(LL), lower limit of 95% bootstrap confidence interval; *CI*(UL), upper limit of 95% bootstrap confidence interval; eZIS, easy Z-score imaging system; NPAs, neuropsychological assessments; MMSE, Mini-Mental State Examination; FAB, Frontal Assessment Battery; MEG, magnetoencephalography; RPa3, relative power in alpha3 band; RPlg, relative power in low gamma band; RPhg, relative power in high gamma band

**Table S7 (cont.). Between-modality correlations between MEG oscillatory parameters averaged across anatomical regions, eZIS parameters, NPAs, and age.**

|  |  | MEG | | | | | | | | |
| --- | --- | --- | --- | --- | --- | --- | --- | --- | --- | --- |
|  |  | MF | | | IAF | | | SSE | | |
|  |  | *rho* | *CI*(LL) | *CI*(UL) | *rho* | *CI*(LL) | *CI*(UL) | *rho* | *CI*(LL) | *CI*(UL) |
| eZIS | *severity* | -0.132 | -0.384 | 0.136 | -0.238 | -0.487 | 0.028 | -0.021 | -0.272 | 0.245 |
|  | *extent* | -0.114 | -0.379 | 0.162 | -0.200 | -0.443 | 0.091 | -0.060 | -0.316 | 0.194 |
|  | *ratio* | -0.060 | -0.309 | 0.198 | -0.172 | -0.415 | 0.096 | 0.010 | -0.240 | 0.260 |
| NPA | MMSE | 0.418* | 0.223 | 0.593 | 0.479* | 0.248 | 0.651 | 0.259* | 0.031 | 0.475 |
|  | FAB | 0.433* | 0.176 | 0.616 | 0.416* | 0.175 | 0.619 | 0.397* | 0.145 | 0.606 |
|  | Age | -0.076 | -0.299 | 0.192 | -0.115 | -0.359 | 0.138 | -0.087 | -0.320 | 0.168 |

*rho*, Spearman’s coefficient, averaged across bootstrap iterations; *CI*(LL), lower limit of 95% bootstrap confidence interval; *CI*(UL), upper limit of 95% bootstrap confidence interval; eZIS, easy Z-score imaging system; NPAs, neuropsychological assessments; MMSE, Mini-Mental State Examination; FAB, Frontal Assessment Battery; MEG, magnetoencephalography; MF, mean frequency; IAF, individual alpha frequency; SSE, Shannon’s spectral entropy

**Table S8. Within- and between-modality correlations among eZIS parameters, NPAs, and age.**

|  |  | eZIS | | | | | | | | |
| --- | --- | --- | --- | --- | --- | --- | --- | --- | --- | --- |
|  |  | *severity* | | | *extent* | | | *ratio* | | |
|  |  | *rho* | *CI*(LL) | *CI*(UL) | *rho* | *CI*(LL) | *CI*(UL) | *rho* | *CI*(LL) | *CI*(UL) |
| NPA | MMSE | 0.003 | -0.267 | 0.277 | 0.016 | -0.261 | 0.284 | 0.216 | -0.047 | 0.448 |
|  | FAB | -0.099 | -0.358 | 0.166 | -0.044 | -0.324 | 0.217 | 0.110 | -0.147 | 0.335 |
|  | Age | -0.096 | -0.374 | 0.183 | -0.164 | -0.438 | 0.121 | -0.339* | -0.555 | -0.055 |

*rho*, Spearman’s coefficient, averaged across bootstrap iterations; *CI*(LL), lower limit of 95% bootstrap confidence interval; *CI*(UL), upper limit of 95% bootstrap confidence interval; eZIS, easy Z-score imaging system; NPAs, neuropsychological assessments; MMSE, Mini-Mental State Examination; FAB, Frontal Assessment Battery

**Table S8 (cont.). Within- and between-modality correlations among eZIS parameters, NPAs, and age.**

|  |  | NPA | | | | | |
| --- | --- | --- | --- | --- | --- | --- | --- |
|  |  | MMSE | | | FAB | | |
|  |  | *rho* | *CI*(LL) | *CI*(UL) | *rho* | *CI*(LL) | *CI*(UL) |
| NPA | FAB | 0.643* | 0.441 | 0.786 |  |  |  |
|  | Age | -0.294* | -0.513 | -0.054 | -0.301* | -0.522 | -0.021 |

*rho*, Spearman’s coefficient, averaged across bootstrap iterations; *CI*(LL), lower limit of 95% bootstrap confidence interval; *CI*(UL), upper limit of 95% bootstrap confidence interval; eZIS, easy Z-score imaging system; NPAs, neuropsychological assessments; MMSE, Mini-Mental State Examination; FAB, Frontal Assessment Battery

# Results of regional correlations between MEG oscillatory and eZIS parameters

**Table S9. Regions and statistical values included in clusters for correlations between MEG oscillatory and eZIS parameters.**

|  |  |  | (A) RPt × *severity* | |  | (B) IAF × *extent* | |
| --- | --- | --- | --- | --- | --- | --- | --- |
| Region | LR |  | *rho* | *T* |  | *rho* | *T* |
| Amygdala | R |  | 0.258 | 2.102 |  |  |  |
| Angular gyrus | L |  | 0.245 | 1.988 |  |  |  |
|  | R |  | 0.345 | 2.890 |  | -0.261 | -2.130 |
| Anterior cingulate cortex-pregenual | L |  | 0.275 | 2.255 |  |  |  |
|  | R |  | 0.239 | 1.936 |  |  |  |
| Anterior cingulate cortex-subgenual | L |  | 0.286 | 2.351 |  |  |  |
|  | R |  | 0.276 | 2.257 |  | -0.258 | -2.098 |
| Anterior cingulate cortex-supracallosal | L |  | 0.250 | 2.029 |  | -0.272 | -2.221 |
|  | R |  | 0.272 | 2.229 |  |  |  |
| Anterior orbital gyrus | L |  | 0.294 | 2.419 |  |  |  |
|  | R |  | 0.276 | 2.262 |  | -0.306 | -2.533 |
| Calcarine fissure and surrounding cortex | L |  | 0.255 | 2.075 |  | -0.264 | -2.156 |
| Crus I of the cerebellar hemisphere | R |  | 0.294 | 2.421 |  | -0.301 | -2.482 |
| Fusiform gyrus | R |  | 0.294 | 2.422 |  | -0.293 | -2.409 |
| Gyrus rectus | R |  | 0.271 | 2.221 |  |  |  |
| Heschl’s gyrus | R |  |  |  |  | -0.252 | -2.052 |
| Hippocampus | R |  |  |  |  | -0.252 | -2.050 |
| IFG pars orbitalis | R |  | 0.263 | 2.149 |  | -0.250 | -2.033 |
| Inferior frontal gyrus-opercular part | R |  | 0.268 | 2.186 |  | -0.265 | -2.161 |
| Inferior frontal gyrus-triangular part | R |  | 0.316 | 2.625 |  | -0.285 | -2.339 |
| Inferior occipital gyrus | R |  | 0.295 | 2.430 |  | -0.288 | -2.371 |
| Inferior parietal gyrus-excluding supramarginal and angular gyri | L |  | 0.283 | 2.323 |  |  |  |
|  | R |  | 0.322 | 2.678 |  | -0.311 | -2.574 |
| Inferior temporal gyrus | R |  |  |  |  | -0.313 | -2.598 |
| Insula | R |  | 0.299 | 2.465 |  |  |  |
| Lateral orbital gyrus | R |  | 0.346 | 2.906 |  | -0.249 | -2.025 |
| Lingual gyrus | L |  |  |  |  | -0.291 | -2.399 |
|  | R |  | 0.252 | 2.054 |  | -0.283 | -2.321 |
| Lobule IV-V of vermis |  |  | 0.252 | 2.053 |  |  |  |
| Lobule VI of cerebellar hemisphere | R |  | 0.291 | 2.396 |  | -0.289 | -2.377 |
| Medial orbital gyrus | L |  | 0.291 | 2.394 |  |  |  |
|  | R |  | 0.297 | 2.453 |  | -0.312 | -2.584 |
| Middle cingulate & paracingulate gyri | R |  | 0.254 | 2.067 |  |  |  |
| Middle frontal gyrus | R |  | 0.261 | 2.125 |  |  |  |
| Middle occipital gyrus | L |  | 0.286 | 2.350 |  |  |  |
| Middle temporal gyrus | R |  | 0.284 | 2.329 |  | -0.260 | -2.122 |
| Olfactory cortex | L |  | 0.256 | 2.087 |  |  |  |
|  | R |  | 0.366 | 3.094 |  | -0.301 | -2.483 |
| Postcentral gyrus | R |  | 0.265 | 2.161 |  | -0.274 | -2.245 |
| Posterior cingulate gyrus | L |  | 0.266 | 2.172 |  |  |  |
|  | R |  | 0.247 | 2.005 |  |  |  |
| Posterior orbital gyrus | L |  | 0.254 | 2.069 |  |  |  |
|  | R |  | 0.254 | 2.070 |  |  |  |
| Precuneus | L |  | 0.264 | 2.154 |  |  |  |
|  | R |  | 0.285 | 2.345 |  |  |  |
| Pulvinar medial | L |  | 0.275 | 2.248 |  |  |  |
| Red nucleus | L |  | 0.272 | 2.230 |  |  |  |
| Substantia nigra-pars compacta | R |  | 0.257 | 2.097 |  |  |  |
| Superior frontal gyrus-dorsolateral | L |  | 0.310 | 2.568 |  | -0.275 | -2.254 |
|  | R |  | 0.271 | 2.213 |  |  |  |
| Superior frontal gyrus-medial | L |  | 0.308 | 2.548 |  | -0.316 | -2.625 |
|  | R |  | 0.310 | 2.565 |  | -0.246 | -2.000 |
| Superior frontal gyrus-medial orbital | L |  | 0.322 | 2.682 |  |  |  |
|  | R |  | 0.257 | 2.097 |  | -0.286 | -2.351 |
| Superior occipital gyrus | R |  | 0.320 | 2.663 |  | -0.255 | -2.073 |
| Superior temporal gyrus | R |  | 0.259 | 2.115 |  | -0.271 | -2.220 |
| SupraMarginal gyrus | R |  | 0.290 | 2.382 |  | -0.294 | -2.424 |
| Mean | |  | 0.281 | 2.310 |  | -0.279 | -2.290 |

**(A) RPt** × ***severity*, and (B) IAF** × ***extent*.** Empty cells indicate the regions are not included in the cluster. RPt, relative power in theta band; IAF, individual alpha frequency; *rho*, Spearman’s coefficient; *T*, *T*-statistic

# Results of regional correlations between MEG oscillatory parameters and MMSE

**Table S10. Regions and statistical values included in clusters for correlations between MEG oscillatory parameters and MMSE**

|  |  |  | (A) RPt × MMSE | |  | (B) RPa1 × MMSE | |  | (C) RPa3 × MMSE | |
| --- | --- | --- | --- | --- | --- | --- | --- | --- | --- | --- |
| Region | LR |  | *rho* | *T* |  | *rho* | *T* |  | *rho* | *T* |
| Amygdala | L |  | -0.424 | -3.691 |  |  |  |  | 0.378 | 3.212 |
|  | R |  | -0.333 | -2.782 |  |  |  |  | 0.268 | 2.193 |
| Angular gyrus | L |  | -0.542 | -5.083 |  | -0.403 | -3.463 |  | 0.540 | 5.048 |
|  | R |  | -0.422 | -3.665 |  |  |  |  | 0.447 | 3.938 |
| Anterior cingulate cortex-supracallosal | L |  | -0.420 | -3.648 |  | -0.361 | -3.047 |  |  |  |
| Anterior orbital gyrus | R |  |  |  |  |  |  |  |  |  |
| Calcarine fissure and surrounding cortex | L |  | -0.264 | -2.157 |  | -0.442 | -3.877 |  |  |  |
|  | R |  | -0.267 | -2.183 |  | -0.416 | -3.598 |  | 0.441 | 3.867 |
| Crus I of the cerebellar hemisphere | L |  | -0.319 | -2.650 |  |  |  |  | 0.367 | 3.103 |
|  | R |  | -0.367 | -3.103 |  |  |  |  | 0.300 | 2.476 |
| Cuneus | L |  | -0.284 | -2.333 |  | -0.403 | -3.468 |  |  |  |
|  | R |  | -0.291 | -2.399 |  | -0.369 | -3.130 |  | 0.377 | 3.205 |
| Fusiform gyrus | L |  | -0.399 | -3.426 |  |  |  |  | 0.343 | 2.875 |
|  | R |  | -0.373 | -3.167 |  |  |  |  | 0.263 | 2.143 |
| Heschl’s gyrus | L |  | -0.543 | -5.096 |  |  |  |  | 0.470 | 4.195 |
|  | R |  | -0.376 | -3.192 |  |  |  |  | 0.395 | 3.383 |
| Hippocampus | L |  | -0.452 | -3.985 |  |  |  |  | 0.395 | 3.382 |
|  | R |  | -0.374 | -3.173 |  |  |  |  | 0.342 | 2.865 |
| Inferior frontal gyrus-opercular part | R |  | -0.298 | -2.456 |  | -0.422 | -3.669 |  |  |  |
| Inferior occipital gyrus | L |  | -0.337 | -2.820 |  | -0.312 | -2.581 |  | 0.268 | 2.192 |
|  | R |  | -0.305 | -2.526 |  | -0.293 | -2.417 |  | 0.266 | 2.171 |
| Inferior parietal gyrus-excluding supramarginal and angular gyri | L |  | -0.444 | -3.897 |  | -0.414 | -3.580 |  | 0.434 | 3.796 |
|  | R |  | -0.398 | -3.414 |  |  |  |  | 0.317 | 2.628 |
| Inferior temporal gyrus | L |  | -0.463 | -4.110 |  |  |  |  | 0.345 | 2.896 |
|  | R |  | -0.324 | -2.698 |  |  |  |  | 0.285 | 2.344 |
| Lingual gyrus | R |  | -0.338 | -2.828 |  |  |  |  | 0.385 | 3.288 |
| Lobule IV-V of the cerebellar hemisphere | L |  | -0.355 | -2.990 |  |  |  |  | 0.343 | 2.878 |
|  | R |  | -0.388 | -3.311 |  |  |  |  | 0.296 | 2.445 |
| Lobule IV-V of vermis |  |  | -0.387 | -3.306 |  |  |  |  | 0.419 | 3.633 |
| Lobule VI of cerebellar hemisphere | L |  | -0.356 | -3.002 |  |  |  |  | 0.256 | 2.088 |
|  | R |  | -0.370 | -3.134 |  | -0.338 | -2.831 |  | 0.471 | 4.204 |
| Mediodorsal medial magnocellular | R |  | -0.418 | -3.618 |  |  |  |  | 0.339 | 2.833 |
| Middle cingulate & paracingulate gyri | L |  | -0.430 | -3.746 |  |  |  |  | 0.352 | 2.960 |
|  | R |  | -0.395 | -3.382 |  | -0.352 | -2.959 |  |  |  |
| Middle frontal gyrus | L |  | -0.308 | -2.545 |  | -0.431 | -3.759 |  |  |  |
|  | R |  | -0.304 | -2.516 |  | -0.472 | -4.219 |  |  |  |
| Middle occipital gyrus | L |  | -0.407 | -3.509 |  | -0.379 | -3.229 |  | 0.472 | 4.218 |
|  | R |  | -0.328 | -2.732 |  |  |  |  | 0.437 | 3.831 |
| Middle temporal gyrus | L |  | -0.507 | -4.632 |  |  |  |  | 0.426 | 3.707 |
|  | R |  | -0.376 | -3.196 |  |  |  |  | 0.469 | 4.186 |
| Paracentral lobule | L |  | -0.374 | -3.180 |  | -0.336 | -2.812 |  |  |  |
|  | R |  | -0.423 | -3.680 |  | -0.392 | -3.354 |  |  |  |
| Parahippocampal gyrus | L |  | -0.378 | -3.218 |  |  |  |  | 0.445 | 3.912 |
|  | R |  | -0.413 | -3.572 |  |  |  |  | 0.388 | 3.319 |
| Postcentral gyrus | L |  | -0.422 | -3.665 |  | -0.414 | -3.584 |  |  |  |
| Posterior cingulate gyrus | L |  | -0.464 | -4.130 |  |  |  |  | 0.398 | 3.413 |
|  | R |  | -0.418 | -3.625 |  |  |  |  | 0.352 | 2.959 |
| Precentral gyrus | L |  | -0.294 | -2.418 |  | -0.355 | -2.993 |  |  |  |
|  | R |  | -0.312 | -2.583 |  | -0.346 | -2.906 |  |  |  |
| Precuneus | L |  | -0.468 | -4.169 |  | -0.418 | -3.624 |  |  |  |
|  | R |  | -0.360 | -3.037 |  | -0.345 | -2.895 |  | 0.395 | 3.388 |
| Pulvinar medial | L |  | -0.384 | -3.279 |  |  |  |  | 0.366 | 3.095 |
| Red nucleus | L |  | -0.420 | -3.645 |  |  |  |  | 0.426 | 3.708 |
| Rolandic operculum | L |  | -0.444 | -3.902 |  |  |  |  | 0.396 | 3.391 |
|  | R |  | -0.367 | -3.107 |  |  |  |  | 0.361 | 3.051 |
| Substantia nigra-pars compacta | L |  | -0.412 | -3.561 |  |  |  |  | 0.453 | 4.001 |
|  | R |  | -0.362 | -3.061 |  |  |  |  | 0.365 | 3.088 |
| Substantia nigra-pars reticulata | R |  | -0.413 | -3.571 |  |  |  |  | 0.333 | 2.777 |
| Superior frontal gyrus-dorsolateral | L |  | -0.356 | -3.003 |  | -0.333 | -2.785 |  |  |  |
|  | R |  | -0.391 | -3.341 |  | -0.394 | -3.380 |  |  |  |
| Superior frontal gyrus-medial | L |  | -0.400 | -3.439 |  | -0.339 | -2.836 |  |  |  |
|  | R |  | -0.378 | -3.215 |  | -0.299 | -2.471 |  |  |  |
| Superior occipital gyrus | L |  | -0.397 | -3.409 |  | -0.339 | -2.839 |  |  |  |
|  | R |  | -0.348 | -2.927 |  | -0.387 | -3.303 |  | 0.401 | 3.442 |
| Superior parietal gyrus | L |  | -0.501 | -4.557 |  | -0.381 | -3.240 |  |  |  |
|  | R |  | -0.474 | -4.236 |  |  |  |  | 0.382 | 3.252 |
| Superior temporal gyrus | L |  | -0.505 | -4.611 |  |  |  |  | 0.430 | 3.749 |
|  | R |  | -0.422 | -3.663 |  |  |  |  | 0.473 | 4.227 |
| Supplementary motor area | L |  | -0.307 | -2.544 |  | -0.358 | -3.022 |  |  |  |
|  | R |  | -0.292 | -2.400 |  | -0.351 | -2.950 |  |  |  |
| SupraMarginal gyrus | L |  | -0.526 | -4.868 |  |  |  |  | 0.473 | 4.224 |
|  | R |  | -0.387 | -3.301 |  |  |  |  | 0.356 | 3.000 |
| Temporal pole: middle temporal gyrus | L |  |  |  |  |  |  |  |  |  |
|  | R |  | -0.311 | -2.578 |  |  |  |  | 0.271 | 2.219 |
| Temporal pole: superior temporal gyrus | L |  | -0.421 | -3.659 |  |  |  |  | 0.301 | 2.487 |
| Mean | |  | -0.387 | -3.332 |  | -0.374 | -3.188 |  | 0.378 | 3.248 |

**(A) RPt × MMSE, (B) RPa1 × MMSE, (C) RPa3 × MMSE.** Empty cells indicate the regions are not included in the cluster. Regions where age showed potential confounding effects (*i.e.* larger variable importance in projection score in partial least square regression than MMSE) were excluded from the list. RPt, relative power in theta band; RPa1, relative power in alpha1 band; RPa3, relative power in alpha3 band; MMSE, Mini-Mental State Examination; *rho*, Spearman’s coefficient; *T*, *T*-statistic.

**Table S11. Regions and statistical values included in clusters for correlations between MEG oscillatory parameters and MMSE.**

|  |  |  | (A) RPb × MMSE | |  | (B) RPlg × MMSE | |
| --- | --- | --- | --- | --- | --- | --- | --- |
| Region | LR |  | *rho* | *T* |  | *rho* | *T* |
| Amygdala | L |  | 0.314 | 2.604 |  |  |  |
|  | R |  | 0.335 | 2.799 |  |  |  |
| Angular gyrus | L |  | 0.572 | 5.489 |  |  |  |
|  | R |  | 0.495 | 4.487 |  | 0.338 | 2.824 |
| Anterior cingulate cortex-pregenual | L |  | 0.379 | 3.222 |  | 0.423 | 3.678 |
|  | R |  | 0.279 | 2.287 |  |  |  |
| Anterior cingulate cortex-subgenual | L |  | 0.375 | 3.182 |  | 0.403 | 3.464 |
|  | R |  | 0.301 | 2.485 |  | 0.279 | 2.286 |
| Anterior cingulate cortex-supracallosal | L |  | 0.381 | 3.242 |  | 0.469 | 4.180 |
|  | R |  | 0.328 | 2.729 |  | 0.484 | 4.356 |
| Anterior orbital gyrus | L |  | 0.270 | 2.212 |  | 0.299 | 2.464 |
|  | R |  | 0.264 | 2.152 |  |  |  |
| Calcarine fissure and surrounding cortex | L |  | 0.423 | 3.675 |  |  |  |
|  | R |  | 0.438 | 3.837 |  |  |  |
| Caudate nucleus | L |  | 0.369 | 3.128 |  | 0.242 | 1.967 |
| Crus I of the cerebellar hemisphere | L |  | 0.412 | 3.565 |  |  |  |
|  | R |  | 0.428 | 3.726 |  |  |  |
| Cuneus | L |  | 0.375 | 3.186 |  |  |  |
|  | R |  | 0.442 | 3.880 |  |  |  |
| Fusiform gyrus | L |  | 0.369 | 3.129 |  |  |  |
|  | R |  | 0.333 | 2.785 |  |  |  |
| Gyrus rectus | L |  | 0.330 | 2.753 |  | 0.388 | 3.317 |
|  | R |  | 0.338 | 2.824 |  | 0.373 | 3.163 |
| Heschl’s gyrus | L |  | 0.468 | 4.165 |  | 0.270 | 2.212 |
|  | R |  | 0.381 | 3.245 |  |  |  |
| Hippocampus | L |  | 0.407 | 3.509 |  |  |  |
|  | R |  | 0.380 | 3.230 |  |  |  |
| IFG pars orbitalis | L |  | 0.258 | 2.101 |  |  |  |
|  | R |  | 0.250 | 2.033 |  |  |  |
| Inferior frontal gyrus-opercular part | L |  | 0.313 | 2.593 |  | 0.431 | 3.764 |
|  | R |  | 0.308 | 2.548 |  | 0.376 | 3.199 |
| Inferior frontal gyrus-triangular part | L |  | 0.299 | 2.464 |  | 0.387 | 3.303 |
|  | R |  |  |  |  | 0.346 | 2.908 |
| Inferior occipital gyrus | L |  | 0.376 | 3.192 |  |  |  |
|  | R |  | 0.359 | 3.033 |  |  |  |
| Inferior parietal gyrus-excluding supramarginal and angular gyri | L |  | 0.527 | 4.880 |  | 0.457 | 4.049 |
|  | R |  | 0.409 | 3.534 |  | 0.344 | 2.885 |
| Inferior temporal gyrus | L |  | 0.441 | 3.874 |  |  |  |
|  | R |  | 0.322 | 2.676 |  |  |  |
| Insula | L |  | 0.311 | 2.579 |  | 0.280 | 2.294 |
|  | R |  | 0.373 | 3.166 |  | 0.259 | 2.115 |
| Lateral orbital gyrus | L |  | 0.407 | 3.507 |  | 0.356 | 2.998 |
|  | R |  | 0.361 | 3.052 |  |  |  |
| Lingual gyrus | L |  | 0.375 | 3.189 |  |  |  |
|  | R |  | 0.349 | 2.936 |  |  |  |
| Lobule IV-V of the cerebellar hemisphere | L |  | 0.431 | 3.765 |  |  |  |
|  | R |  | 0.367 | 3.111 |  |  |  |
| Lobule IV-V of vermis |  |  | 0.368 | 3.116 |  |  |  |
| Lobule VI of cerebellar hemisphere | L |  | 0.322 | 2.682 |  |  |  |
|  | R |  | 0.460 | 4.075 |  |  |  |
| Medial orbital gyrus | L |  | 0.309 | 2.561 |  | 0.271 | 2.221 |
|  | R |  | 0.311 | 2.580 |  |  |  |
| Mediodorsal medial magnocellular | R |  | 0.464 | 4.121 |  | 0.325 | 2.703 |
| Middle cingulate & paracingulate gyri | L |  | 0.328 | 2.733 |  | 0.403 | 3.462 |
|  | R |  | 0.469 | 4.185 |  | 0.476 | 4.261 |
| Middle frontal gyrus | L |  |  |  |  | 0.473 | 4.227 |
|  | R |  | 0.258 | 2.102 |  | 0.391 | 3.350 |
| Middle occipital gyrus | L |  | 0.538 | 5.022 |  | 0.282 | 2.314 |
|  | R |  | 0.463 | 4.112 |  |  |  |
| Middle temporal gyrus | L |  | 0.474 | 4.238 |  |  |  |
|  | R |  | 0.458 | 4.056 |  |  |  |
| Olfactory cortex | L |  | 0.315 | 2.614 |  | 0.336 | 2.805 |
|  | R |  | 0.284 | 2.333 |  | 0.314 | 2.602 |
| Paracentral lobule | R |  | 0.311 | 2.576 |  |  |  |
| Parahippocampal gyrus | L |  | 0.417 | 3.618 |  |  |  |
|  | R |  | 0.380 | 3.237 |  |  |  |
| Postcentral gyrus | L |  | 0.385 | 3.289 |  | 0.377 | 3.204 |
|  | R |  | 0.345 | 2.895 |  | 0.297 | 2.448 |
| Posterior cingulate gyrus | L |  | 0.527 | 4.879 |  | 0.317 | 2.630 |
|  | R |  | 0.336 | 2.813 |  |  |  |
| Posterior orbital gyrus | L |  | 0.258 | 2.107 |  |  |  |
|  | R |  | 0.246 | 1.999 |  |  |  |
| Precentral gyrus | L |  |  |  |  | 0.465 | 4.137 |
|  | R |  | 0.271 | 2.217 |  | 0.388 | 3.316 |
| Precuneus | L |  | 0.528 | 4.898 |  | 0.273 | 2.237 |
|  | R |  | 0.442 | 3.884 |  |  |  |
| Pulvinar medial | L |  | 0.321 | 2.672 |  |  |  |
| Red nucleus | L |  | 0.414 | 3.584 |  |  |  |
| Rolandic operculum | L |  | 0.363 | 3.066 |  |  |  |
|  | R |  | 0.341 | 2.859 |  |  |  |
| Substantia nigra-pars compacta | L |  | 0.466 | 4.147 |  |  |  |
|  | R |  | 0.355 | 2.993 |  |  |  |
| Substantia nigra-pars reticulata | R |  | 0.301 | 2.486 |  |  |  |
| Superior frontal gyrus-dorsolateral | L |  | 0.281 | 2.305 |  | 0.433 | 3.779 |
|  | R |  | 0.322 | 2.674 |  | 0.396 | 3.392 |
| Superior frontal gyrus-medial | L |  | 0.287 | 2.358 |  | 0.432 | 3.769 |
|  | R |  | 0.285 | 2.345 |  | 0.401 | 3.448 |
| Superior frontal gyrus-medial orbital | L |  | 0.318 | 2.638 |  | 0.360 | 3.042 |
|  | R |  | 0.277 | 2.270 |  |  |  |
| Superior occipital gyrus | L |  | 0.399 | 3.426 |  |  |  |
|  | R |  | 0.456 | 4.032 |  |  |  |
| Superior parietal gyrus | L |  | 0.544 | 5.108 |  | 0.340 | 2.844 |
|  | R |  | 0.504 | 4.595 |  | 0.309 | 2.554 |
| Superior temporal gyrus | L |  | 0.475 | 4.249 |  | 0.291 | 2.399 |
|  | R |  | 0.386 | 3.299 |  |  |  |
| Supplementary motor area | L |  |  |  |  | 0.452 | 3.986 |
|  | R |  |  |  |  | 0.442 | 3.883 |
| SupraMarginal gyrus | L |  | 0.521 | 4.806 |  | 0.416 | 3.603 |
|  | R |  | 0.393 | 3.367 |  |  |  |
| Temporal pole: middle temporal gyrus | L |  | 0.262 | 2.139 |  |  |  |
|  | R |  | 0.274 | 2.240 |  |  |  |
| Temporal pole: superior temporal gyrus | L |  | 0.405 | 3.486 |  | 0.310 | 2.569 |
|  | R |  | 0.268 | 2.188 |  |  |  |
| Mean | |  | 0.373 | 3.204 |  | 0.365 | 3.119 |

**(A) RPb × MMSE, (B) RPlg × MMSE.** Empty cells indicate the regions are not included in the cluster. Regions where age showed potential confounding effects (*i.e.* larger variable importance in projection score in partial least square regression than MMSE) were excluded from the list. RPb, relative power in beta band; RPlg, relative power in low gamma band; MMSE, Mini-Mental State Examination; *rho*, Spearman’s coefficient; *T*, *T*-statistic

**Table S12. Regions and statistical values included in clusters for correlations between MEG oscillatory parameters and MMSE.**

|  |  |  | (A) MF × MMSE | |  | (B) IAF × MMSE | |  | (C) SSE × MMSE | |
| --- | --- | --- | --- | --- | --- | --- | --- | --- | --- | --- |
| Region | LR |  | *rho* | *T* |  | *rho* | *T* |  | *rho* | *T* |
| Amygdala | L |  | 0.399 | 3.431 |  | 0.510 | 4.670 |  |  |  |
|  | R |  | 0.296 | 2.444 |  | 0.430 | 3.752 |  |  |  |
| Angular gyrus | L |  | 0.599 | 5.896 |  | 0.611 | 6.076 |  | 0.409 | 3.533 |
|  | R |  | 0.490 | 4.421 |  | 0.518 | 4.774 |  | 0.289 | 2.375 |
| Anterior cingulate cortex-pregenual | L |  | 0.367 | 3.103 |  | 0.380 | 3.236 |  | 0.340 | 2.842 |
| Anterior cingulate cortex-subgenual | L |  | 0.292 | 2.404 |  | 0.443 | 3.890 |  | 0.328 | 2.732 |
| Anterior cingulate cortex-supracallosal | L |  | 0.403 | 3.466 |  | 0.385 | 3.288 |  | 0.371 | 3.146 |
|  | R |  | 0.450 | 3.969 |  | 0.379 | 3.223 |  | 0.475 | 4.250 |
| Anterior orbital gyrus | L |  |  |  |  |  |  |  |  |  |
| Calcarine fissure and surrounding cortex | L |  | 0.322 | 2.674 |  | 0.353 | 2.968 |  |  |  |
|  | R |  | 0.311 | 2.574 |  | 0.374 | 3.178 |  |  |  |
| Caudate nucleus | L |  | 0.302 | 2.496 |  | 0.519 | 4.785 |  |  |  |
| Crus I of the cerebellar hemisphere | L |  | 0.373 | 3.162 |  | 0.386 | 3.299 |  |  |  |
|  | R |  | 0.320 | 2.663 |  | 0.401 | 3.444 |  | 0.239 | 1.939 |
| Cuneus | L |  | 0.350 | 2.942 |  | 0.332 | 2.774 |  |  |  |
|  | R |  | 0.347 | 2.916 |  | 0.336 | 2.807 |  |  |  |
| Fusiform gyrus | L |  | 0.297 | 2.446 |  | 0.485 | 4.363 |  |  |  |
| Gyrus rectus | L |  | 0.255 | 2.072 |  | 0.389 | 3.324 |  |  |  |
|  | R |  | 0.276 | 2.264 |  | 0.424 | 3.687 |  |  |  |
| Heschl’s gyrus | L |  | 0.515 | 4.731 |  | 0.581 | 5.614 |  | 0.307 | 2.540 |
|  | R |  | 0.353 | 2.971 |  | 0.478 | 4.287 |  |  |  |
| Hippocampus | L |  | 0.331 | 2.761 |  | 0.504 | 4.599 |  |  |  |
|  | R |  | 0.306 | 2.535 |  | 0.485 | 4.362 |  |  |  |
| Inferior frontal gyrus-opercular part | L |  | 0.386 | 3.297 |  | 0.317 | 2.635 |  | 0.441 | 3.872 |
|  | R |  | 0.363 | 3.064 |  | 0.302 | 2.493 |  | 0.334 | 2.789 |
| Inferior frontal gyrus-triangular part | L |  | 0.291 | 2.393 |  | 0.352 | 2.957 |  | 0.285 | 2.341 |
|  | R |  | 0.347 | 2.913 |  | 0.287 | 2.357 |  | 0.264 | 2.155 |
| Inferior occipital gyrus | L |  | 0.349 | 2.937 |  | 0.347 | 2.915 |  |  |  |
|  | R |  | 0.397 | 3.409 |  | 0.332 | 2.768 |  | 0.267 | 2.178 |
| Inferior parietal gyrus-excluding supramarginal and angular gyri | L |  | 0.534 | 4.968 |  | 0.517 | 4.755 |  | 0.445 | 3.916 |
|  | R |  | 0.381 | 3.248 |  | 0.423 | 3.677 |  | 0.268 | 2.186 |
| Inferior temporal gyrus | L |  | 0.262 | 2.134 |  | 0.526 | 4.865 |  |  |  |
| Insula | L |  | 0.289 | 2.377 |  | 0.485 | 4.362 |  |  |  |
|  | R |  | 0.307 | 2.537 |  | 0.389 | 3.327 |  | 0.288 | 2.364 |
| Lateral orbital gyrus | L |  | 0.396 | 3.399 |  | 0.349 | 2.934 |  | 0.344 | 2.883 |
|  | R |  | 0.284 | 2.333 |  | 0.279 | 2.286 |  |  |  |
| Lingual gyrus | L |  | 0.261 | 2.129 |  | 0.394 | 3.378 |  |  |  |
| Lobule IV-V of the cerebellar hemisphere | L |  | 0.332 | 2.767 |  | 0.439 | 3.845 |  |  |  |
|  | R |  | 0.283 | 2.322 |  | 0.482 | 4.336 |  |  |  |
| Lobule IV-V of vermis |  |  | 0.349 | 2.935 |  | 0.424 | 3.684 |  |  |  |
| Lobule VI of cerebellar hemisphere | L |  | 0.288 | 2.366 |  | 0.382 | 3.253 |  |  |  |
|  | R |  | 0.362 | 3.054 |  | 0.443 | 3.887 |  |  |  |
| Mediodorsal medial magnocellular | R |  | 0.421 | 3.659 |  | 0.495 | 4.491 |  |  |  |
| Middle cingulate & paracingulate gyri | L |  | 0.428 | 3.726 |  | 0.451 | 3.977 |  | 0.401 | 3.452 |
|  | R |  | 0.494 | 4.474 |  | 0.441 | 3.870 |  | 0.452 | 3.994 |
| Middle frontal gyrus | L |  | 0.362 | 3.058 |  |  |  |  | 0.331 | 2.758 |
|  | R |  | 0.367 | 3.111 |  | 0.266 | 2.172 |  | 0.271 | 2.213 |
| Middle occipital gyrus | L |  | 0.489 | 4.415 |  | 0.490 | 4.428 |  | 0.307 | 2.541 |
|  | R |  | 0.382 | 3.259 |  | 0.368 | 3.113 |  |  |  |
| Middle temporal gyrus | L |  | 0.429 | 3.738 |  | 0.524 | 4.844 |  | 0.265 | 2.166 |
|  | R |  | 0.437 | 3.827 |  | 0.485 | 4.364 |  | 0.254 | 2.069 |
| Olfactory cortex | L |  | 0.272 | 2.223 |  | 0.467 | 4.163 |  |  |  |
|  | R |  |  |  |  | 0.311 | 2.579 |  | 0.251 | 2.041 |
| Paracentral lobule | L |  | 0.410 | 3.544 |  | 0.320 | 2.655 |  | 0.333 | 2.778 |
|  | R |  | 0.433 | 3.787 |  | 0.435 | 3.800 |  |  |  |
| Parahippocampal gyrus | L |  | 0.335 | 2.804 |  | 0.526 | 4.871 |  |  |  |
|  | R |  | 0.369 | 3.129 |  | 0.518 | 4.773 |  | 0.239 | 1.936 |
| Postcentral gyrus | L |  | 0.476 | 4.264 |  | 0.491 | 4.432 |  | 0.347 | 2.909 |
|  | R |  | 0.375 | 3.189 |  | 0.350 | 2.940 |  | 0.256 | 2.084 |
| Posterior cingulate gyrus | L |  | 0.502 | 4.574 |  | 0.498 | 4.527 |  | 0.346 | 2.900 |
|  | R |  | 0.272 | 2.228 |  | 0.429 | 3.744 |  |  |  |
| Precentral gyrus | L |  | 0.363 | 3.065 |  | 0.295 | 2.428 |  | 0.348 | 2.927 |
|  | R |  | 0.342 | 2.868 |  | 0.306 | 2.532 |  | 0.305 | 2.519 |
| Precuneus | L |  | 0.550 | 5.191 |  | 0.476 | 4.264 |  | 0.258 | 2.101 |
|  | R |  | 0.395 | 3.385 |  | 0.399 | 3.428 |  |  |  |
| Pulvinar medial | L |  | 0.256 | 2.085 |  | 0.464 | 4.124 |  |  |  |
| Red nucleus | L |  | 0.344 | 2.881 |  | 0.501 | 4.557 |  |  |  |
| Rolandic operculum | L |  | 0.362 | 3.062 |  | 0.464 | 4.124 |  | 0.245 | 1.991 |
|  | R |  | 0.382 | 3.253 |  | 0.485 | 4.362 |  | 0.328 | 2.738 |
| Substantia nigra-pars compacta | L |  | 0.509 | 4.651 |  | 0.512 | 4.699 |  | 0.299 | 2.470 |
|  | R |  | 0.345 | 2.899 |  | 0.438 | 3.839 |  |  |  |
| Substantia nigra-pars reticulata | R |  | 0.307 | 2.539 |  | 0.452 | 3.995 |  |  |  |
| Superior frontal gyrus-dorsolateral | L |  | 0.358 | 3.020 |  | 0.291 | 2.396 |  | 0.336 | 2.812 |
|  | R |  | 0.366 | 3.099 |  | 0.349 | 2.932 |  | 0.286 | 2.353 |
| Superior frontal gyrus-medial | L |  | 0.363 | 3.069 |  | 0.307 | 2.540 |  | 0.305 | 2.523 |
|  | R |  | 0.354 | 2.980 |  | 0.331 | 2.762 |  | 0.267 | 2.183 |
| Superior frontal gyrus-medial orbital | L |  | 0.261 | 2.133 |  | 0.298 | 2.461 |  | 0.254 | 2.066 |
| Superior occipital gyrus | L |  | 0.424 | 3.686 |  | 0.403 | 3.466 |  |  |  |
|  | R |  | 0.425 | 3.701 |  | 0.406 | 3.497 |  |  |  |
| Superior parietal gyrus | L |  | 0.553 | 5.223 |  | 0.481 | 4.317 |  | 0.309 | 2.555 |
|  | R |  | 0.517 | 4.760 |  | 0.507 | 4.633 |  | 0.274 | 2.243 |
| Superior temporal gyrus | L |  | 0.481 | 4.316 |  | 0.532 | 4.948 |  | 0.299 | 2.466 |
|  | R |  | 0.404 | 3.474 |  | 0.477 | 4.268 |  |  |  |
| Supplementary motor area | L |  | 0.347 | 2.917 |  |  |  |  | 0.318 | 2.639 |
|  | R |  | 0.356 | 2.996 |  | 0.241 | 1.954 |  | 0.329 | 2.746 |
| SupraMarginal gyrus | L |  | 0.518 | 4.769 |  | 0.552 | 5.206 |  | 0.389 | 3.322 |
|  | R |  | 0.375 | 3.184 |  | 0.476 | 4.259 |  |  |  |
| Temporal pole: superior temporal gyrus | L |  | 0.258 | 2.102 |  | 0.473 | 4.224 |  | 0.275 | 2.250 |
|  | R |  | 0.292 | 2.408 |  | 0.434 | 3.797 |  |  |  |
| Mean | |  | 0.373 | 3.214 |  | 0.423 | 3.731 |  | 0.316 | 2.641 |

**(A) MF × MMSE, (B) IAF × MMSE, (C) SSE × MMSE.** Empty cells indicate the regions are not included in the cluster. Regions where age showed potential confounding effects (*i.e.* larger variable importance in projection score in partial least square regression than MMSE) were excluded from the list. MF, mean frequency; IAF, individual alpha frequency; SSE, Shannon’s spectral entropy; MMSE, Mini-Mental State Examination; *rho*, Spearman’s coefficient; *T*, *T*-statistic

# Results of regional correlations between MEG oscillatory parameters and FAB

**Table S13. Regions and statistical values included in clusters for correlations between MEG oscillatory parameters and FAB.**

|  |  |  | (A) RPt × FAB | |  | (B) RPa1 × FAB | |  | (C) RPa3 × FAB | |
| --- | --- | --- | --- | --- | --- | --- | --- | --- | --- | --- |
| Region | LR |  | *rho* | *T* |  | *rho* | *T* |  | *rho* | *T* |
| Amygdala | L |  | -0.341 | -2.832 |  |  |  |  | 0.379 | 3.200 |
|  | R |  | -0.330 | -2.734 |  |  |  |  | 0.284 | 2.314 |
| Angular gyrus | L |  | -0.448 | -3.916 |  | -0.308 | -2.530 |  | 0.619 | 6.156 |
|  | R |  | -0.377 | -3.181 |  |  |  |  | 0.451 | 3.951 |
| Anterior orbital gyrus | L |  |  |  |  |  |  |  |  |  |
| Calcarine fissure and surrounding cortex | L |  |  |  |  | -0.357 | -2.985 |  | 0.271 | 2.196 |
|  | R |  |  |  |  | -0.381 | -3.223 |  | 0.320 | 2.639 |
| Crus I of the cerebellar hemisphere | R |  | -0.348 | -2.897 |  |  |  |  | 0.277 | 2.249 |
| Cuneus | R |  |  |  |  | -0.369 | -3.105 |  | 0.387 | 3.283 |
| Fusiform gyrus | L |  | -0.266 | -2.152 |  |  |  |  | 0.515 | 4.695 |
| Gyrus rectus | L |  |  |  |  |  |  |  |  |  |
| Heschl’s gyrus | L |  | -0.460 | -4.045 |  |  |  |  | 0.579 | 5.548 |
|  | R |  | -0.387 | -3.278 |  |  |  |  | 0.512 | 4.653 |
| Hippocampus | L |  | -0.372 | -3.129 |  |  |  |  | 0.496 | 4.462 |
|  | R |  | -0.354 | -2.960 |  |  |  |  | 0.399 | 3.403 |
| Inferior frontal gyrus-opercular part | R |  | -0.305 | -2.501 |  | -0.374 | -3.149 |  |  |  |
| Inferior occipital gyrus | L |  | -0.271 | -2.202 |  | -0.289 | -2.355 |  | 0.295 | 2.408 |
| Inferior parietal gyrus-excluding supramarginal and angular gyri | L |  | -0.394 | -3.346 |  | -0.276 | -2.244 |  | 0.499 | 4.493 |
|  | R |  | -0.319 | -2.633 |  |  |  |  | 0.344 | 2.859 |
| Inferior temporal gyrus | L |  | -0.282 | -2.294 |  |  |  |  | 0.474 | 4.200 |
|  | R |  | -0.264 | -2.139 |  |  |  |  | 0.359 | 3.007 |
| Insula | L |  | -0.314 | -2.586 |  |  |  |  | 0.284 | 2.317 |
| Lingual gyrus | R |  | -0.256 | -2.068 |  | -0.311 | -2.552 |  |  |  |
| Lobule IV-V of the cerebellar hemisphere | R |  | -0.344 | -2.864 |  |  |  |  | 0.355 | 2.964 |
| Lobule IV-V of vermis |  |  | -0.299 | -2.446 |  |  |  |  | 0.311 | 2.559 |
| Lobule VI of cerebellar hemisphere | R |  | -0.350 | -2.920 |  | -0.385 | -3.256 |  | 0.318 | 2.622 |
| Medial orbital gyrus | L |  |  |  |  |  |  |  |  |  |
| Mediodorsal medial magnocellular | R |  | -0.376 | -3.173 |  |  |  |  | 0.419 | 3.606 |
| Middle cingulate & paracingulate gyri | L |  | -0.400 | -3.407 |  |  |  |  | 0.306 | 2.510 |
|  | R |  | -0.352 | -2.933 |  | -0.315 | -2.594 |  |  |  |
| Middle frontal gyrus | R |  | -0.283 | -2.302 |  | -0.444 | -3.871 |  |  |  |
| Middle occipital gyrus | L |  | -0.358 | -2.998 |  | -0.334 | -2.771 |  | 0.558 | 5.258 |
|  | R |  | -0.305 | -2.498 |  |  |  |  | 0.478 | 4.255 |
| Middle temporal gyrus | L |  | -0.424 | -3.661 |  |  |  |  | 0.567 | 5.373 |
|  | R |  | -0.344 | -2.863 |  |  |  |  | 0.435 | 3.776 |
| Olfactory cortex | R |  |  |  |  |  |  |  |  |  |
| Paracentral lobule | L |  | -0.253 | -2.044 |  | -0.270 | -2.187 |  |  |  |
|  | R |  | -0.308 | -2.529 |  | -0.346 | -2.884 |  |  |  |
| Parahippocampal gyrus | L |  | -0.329 | -2.719 |  |  |  |  | 0.544 | 5.061 |
|  | R |  | -0.366 | -3.070 |  |  |  |  | 0.456 | 4.003 |
| Postcentral gyrus | L |  | -0.340 | -2.824 |  |  |  |  | 0.286 | 2.327 |
| Posterior cingulate gyrus | L |  | -0.434 | -3.760 |  |  |  |  | 0.425 | 3.663 |
|  | R |  | -0.268 | -2.170 |  |  |  |  | 0.284 | 2.311 |
| Posterior orbital gyrus | L |  |  |  |  |  |  |  |  |  |
|  | R |  |  |  |  |  |  |  |  |  |
| Precuneus | L |  | -0.305 | -2.501 |  | -0.408 | -3.490 |  |  |  |
|  | R |  | -0.268 | -2.173 |  | -0.385 | -3.262 |  | 0.370 | 3.113 |
| Pulvinar medial | L |  | -0.285 | -2.326 |  |  |  |  | 0.450 | 3.936 |
| Red nucleus | L |  | -0.297 | -2.428 |  |  |  |  | 0.476 | 4.231 |
| Rolandic operculum | L |  | -0.448 | -3.914 |  |  |  |  | 0.451 | 3.951 |
|  | R |  | -0.346 | -2.885 |  |  |  |  | 0.445 | 3.885 |
| Substantia nigra-pars compacta | L |  | -0.313 | -2.575 |  |  |  |  | 0.421 | 3.623 |
|  | R |  | -0.336 | -2.783 |  |  |  |  | 0.445 | 3.876 |
| Substantia nigra-pars reticulata | R |  | -0.356 | -2.976 |  |  |  |  | 0.428 | 3.698 |
| Superior frontal gyrus-dorsolateral | L |  | -0.341 | -2.832 |  | -0.286 | -2.331 |  |  |  |
|  | R |  | -0.362 | -3.029 |  | -0.321 | -2.646 |  |  |  |
| Superior frontal gyrus-medial | L |  | -0.393 | -3.333 |  | -0.336 | -2.785 |  |  |  |
|  | R |  | -0.377 | -3.181 |  | -0.307 | -2.522 |  |  |  |
| Superior occipital gyrus | L |  | -0.328 | -2.715 |  | -0.370 | -3.111 |  |  |  |
|  | R |  | -0.331 | -2.739 |  | -0.382 | -3.229 |  | 0.406 | 3.474 |
| Superior parietal gyrus | L |  | -0.349 | -2.910 |  | -0.378 | -3.191 |  |  |  |
|  | R |  | -0.332 | -2.745 |  | -0.372 | -3.126 |  | 0.359 | 3.009 |
| Superior temporal gyrus | L |  | -0.490 | -4.395 |  |  |  |  | 0.556 | 5.218 |
|  | R |  | -0.396 | -3.365 |  |  |  |  | 0.564 | 5.328 |
| Supplementary motor area | L |  | -0.352 | -2.935 |  | -0.323 | -2.665 |  |  |  |
|  | R |  | -0.309 | -2.533 |  | -0.337 | -2.799 |  |  |  |
| SupraMarginal gyrus | L |  | -0.477 | -4.238 |  |  |  |  | 0.605 | 5.931 |
|  | R |  | -0.338 | -2.800 |  |  |  |  | 0.321 | 2.648 |
| Temporal pole: middle temporal gyrus | L |  |  |  |  |  |  |  |  |  |
| Temporal pole: superior temporal gyrus | L |  | -0.346 | -2.883 |  |  |  |  | 0.332 | 2.751 |
| Mean | |  | -0.345 | -2.888 |  | -0.345 | -2.879 |  | 0.419 | 3.687 |

**(A) RPt × FAB, (B) RPa1 × FAB, (C) RPa3 × FAB.** Empty cells indicate the regions are not included in the cluster. Regions where age showed potential confounding effects (*i.e.* larger variable importance in projection score in partial least square regression than FAB) were excluded from the list. RPt, relative power in theta band; RPa1, relative power in alpha1 band; RPa3, relative power in alpha3 band; FAB, Frontal Assessment Battery; *rho*, Spearman’s coefficient; *T*, *T*-statistic

**Table S14. Regions and statistical values included in clusters for correlations between MEG oscillatory parameters and FAB.**

|  |  |  | (A) RPb × FAB | |  | (B) RPlg × FAB | |  | (C) RPhg × FAB | |
| --- | --- | --- | --- | --- | --- | --- | --- | --- | --- | --- |
| Region | LR |  | *rho* | *T* |  | *rho* | *T* |  | *rho* | *T* |
| Amygdala | L |  | 0.289 | 2.357 |  | 0.258 | 2.085 |  |  |  |
|  | R |  | 0.280 | 2.282 |  | 0.252 | 2.030 |  |  |  |
| Angular gyrus | L |  | 0.475 | 4.213 |  | 0.502 | 4.530 |  |  |  |
|  | R |  | 0.435 | 3.771 |  | 0.508 | 4.611 |  | 0.441 | 3.833 |
| Anterior cingulate cortex-pregenual | L |  | 0.293 | 2.393 |  | 0.500 | 4.513 |  | 0.447 | 3.900 |
|  | R |  |  |  |  |  |  |  |  |  |
| Anterior cingulate cortex-subgenual | L |  | 0.282 | 2.296 |  | 0.393 | 3.334 |  | 0.299 | 2.443 |
|  | R |  | 0.309 | 2.539 |  | 0.395 | 3.361 |  | 0.309 | 2.534 |
| Anterior cingulate cortex-supracallosal | L |  | 0.330 | 2.728 |  | 0.517 | 4.719 |  |  |  |
|  | R |  | 0.304 | 2.492 |  | 0.496 | 4.467 |  |  |  |
| Anterior orbital gyrus | R |  | 0.270 | 2.192 |  | 0.281 | 2.284 |  |  |  |
| Calcarine fissure and surrounding cortex | R |  | 0.376 | 3.173 |  | 0.253 | 2.042 |  |  |  |
| Caudate nucleus | L |  | 0.330 | 2.732 |  | 0.298 | 2.439 |  |  |  |
| Crus I of the cerebellar hemisphere | L |  | 0.396 | 3.369 |  | 0.320 | 2.637 |  |  |  |
|  | R |  | 0.302 | 2.475 |  | 0.350 | 2.922 |  |  |  |
| Cuneus | R |  | 0.413 | 3.544 |  | 0.265 | 2.149 |  | 0.245 | 1.978 |
| Fusiform gyrus | L |  | 0.435 | 3.771 |  | 0.249 | 2.006 |  |  |  |
|  | R |  |  |  |  |  |  |  |  |  |
| Gyrus rectus | L |  |  |  |  | 0.391 | 3.321 |  | 0.330 | 2.727 |
|  | R |  | 0.293 | 2.393 |  | 0.424 | 3.660 |  | 0.383 | 3.243 |
| Heschl’s gyrus | L |  | 0.439 | 3.816 |  | 0.292 | 2.387 |  |  |  |
| Inferior frontal gyrus-opercular part | L |  | 0.248 | 2.002 |  | 0.455 | 3.993 |  |  |  |
|  | R |  | 0.252 | 2.035 |  | 0.499 | 4.501 |  |  |  |
| Inferior frontal gyrus-triangular part | R |  |  |  |  |  |  |  |  |  |
| Inferior occipital gyrus | L |  | 0.363 | 3.044 |  | 0.326 | 2.690 |  |  |  |
|  | R |  | 0.301 | 2.468 |  | 0.397 | 3.382 |  | 0.304 | 2.494 |
| Inferior parietal gyrus-excluding supramarginal and angular gyri | L |  | 0.430 | 3.718 |  | 0.503 | 4.548 |  |  |  |
|  | R |  | 0.352 | 2.938 |  | 0.438 | 3.805 |  |  |  |
| Insula | L |  | 0.303 | 2.482 |  | 0.368 | 3.093 |  |  |  |
|  | R |  | 0.310 | 2.549 |  | 0.409 | 3.500 |  |  |  |
| Lateral orbital gyrus | L |  | 0.339 | 2.819 |  | 0.366 | 3.073 |  |  |  |
|  | R |  | 0.378 | 3.193 |  | 0.426 | 3.680 |  |  |  |
| Lingual gyrus | R |  |  |  |  |  |  |  |  |  |
| Lobule IV-V of the cerebellar hemisphere | L |  | 0.471 | 4.174 |  | 0.279 | 2.266 |  |  |  |
| Lobule IV-V of vermis |  |  | 0.290 | 2.368 |  | 0.262 | 2.124 |  |  |  |
| Lobule VI of the cerebellar hemisphere | L |  | 0.344 | 2.861 |  | 0.285 | 2.322 |  |  |  |
| Medial orbital gyrus | L |  | 0.260 | 2.105 |  | 0.314 | 2.583 |  |  |  |
|  | R |  | 0.318 | 2.619 |  | 0.289 | 2.354 |  |  |  |
| Mediodorsal medial magnocellular | R |  | 0.401 | 3.417 |  | 0.393 | 3.340 |  |  |  |
| Middle cingulate & paracingulate gyri | L |  | 0.271 | 2.198 |  | 0.444 | 3.867 |  |  |  |
|  | R |  | 0.366 | 3.069 |  | 0.506 | 4.586 |  |  |  |
| Middle frontal gyrus | R |  | 0.256 | 2.070 |  | 0.443 | 3.859 |  |  |  |
| Middle occipital gyrus | L |  | 0.461 | 4.057 |  | 0.326 | 2.698 |  |  |  |
|  | R |  | 0.489 | 4.379 |  | 0.374 | 3.152 |  | 0.373 | 3.143 |
| Middle temporal gyrus | L |  | 0.464 | 4.093 |  | 0.272 | 2.210 |  |  |  |
|  | R |  | 0.403 | 3.434 |  | 0.288 | 2.346 |  |  |  |
| Olfactory cortex | L |  | 0.306 | 2.507 |  | 0.402 | 3.430 |  |  |  |
|  | R |  | 0.317 | 2.606 |  | 0.379 | 3.203 |  |  |  |
| Paracentral lobule | R |  |  |  |  |  |  |  |  |  |
| Parahippocampal gyrus | R |  | 0.318 | 2.624 |  | 0.323 | 2.661 |  |  |  |
| Postcentral gyrus | L |  | 0.354 | 2.959 |  | 0.393 | 3.343 |  |  |  |
|  | R |  | 0.331 | 2.742 |  | 0.327 | 2.698 |  |  |  |
| Posterior cingulate gyrus | L |  | 0.473 | 4.194 |  | 0.428 | 3.695 |  |  |  |
| Posterior orbital gyrus | L |  |  |  |  |  |  |  |  |  |
| Precuneus | L |  | 0.391 | 3.314 |  | 0.367 | 3.082 |  | 0.293 | 2.391 |
|  | R |  | 0.403 | 3.442 |  | 0.302 | 2.476 |  |  |  |
| Substantia nigra-pars compacta | R |  | 0.350 | 2.917 |  | 0.310 | 2.543 |  |  |  |
| Substantia nigra-pars reticulata | R |  | 0.275 | 2.231 |  | 0.266 | 2.158 |  |  |  |
| Superior frontal gyrus-dorsolateral | L |  |  |  |  | 0.479 | 4.267 |  | 0.336 | 2.785 |
|  | R |  |  |  |  | 0.458 | 4.026 |  | 0.344 | 2.863 |
| Superior frontal gyrus-medial | L |  | 0.257 | 2.075 |  | 0.478 | 4.252 |  | 0.431 | 3.726 |
|  | R |  | 0.256 | 2.066 |  | 0.467 | 4.128 |  | 0.419 | 3.603 |
| Superior frontal gyrus-medial orbital | L |  | 0.289 | 2.353 |  | 0.354 | 2.961 |  | 0.271 | 2.202 |
|  | R |  | 0.276 | 2.241 |  | 0.255 | 2.055 |  |  |  |
| Superior occipital gyrus | L |  | 0.308 | 2.527 |  | 0.253 | 2.042 |  | 0.270 | 2.188 |
|  | R |  | 0.439 | 3.812 |  | 0.369 | 3.103 |  | 0.364 | 3.051 |
| Superior parietal gyrus | L |  | 0.383 | 3.239 |  | 0.427 | 3.692 |  | 0.372 | 3.130 |
|  | R |  | 0.455 | 3.996 |  | 0.375 | 3.161 |  | 0.356 | 2.977 |
| Superior temporal gyrus | L |  | 0.483 | 4.307 |  | 0.281 | 2.287 |  |  |  |
| SupraMarginal gyrus | L |  | 0.514 | 4.678 |  | 0.486 | 4.344 |  |  |  |
|  | R |  | 0.403 | 3.444 |  | 0.362 | 3.032 |  |  |  |
| Temporal pole: middle temporal gyrus | L |  | 0.249 | 2.009 |  | 0.314 | 2.581 |  |  |  |
| Temporal pole: superior temporal gyrus | L |  | 0.336 | 2.789 |  | 0.366 | 3.071 |  |  |  |
| Mean | |  | 0.351 | 2.964 |  | 0.371 | 3.161 |  | 0.347 | 2.906 |

**(A) RPb × FAB, (B) RPlg × FAB, (C) RPhg × FAB.** Empty cells indicate the regions are not included in the cluster. Regions where age showed potential confounding effects (*i.e.* larger variable importance in projection score in partial least square regression than FAB), were excluded from the list. RPb, relative power in beta band; RPlg, relative power in low gamma band; RPhg, relative power in high gamma band; FAB, Frontal Assessment Battery; *rho*, Spearman’s coefficient; *T*, *T*-statistic

**Table S15. Regions and statistical values included in clusters for correlations between MEG oscillatory parameters and FAB.**

|  |  |  | (A) MF × FAB | |  | (B) IAF × FAB | |  | (C) SSE × FAB | |
| --- | --- | --- | --- | --- | --- | --- | --- | --- | --- | --- |
| Region | LR |  | *rho* | *T* |  | *rho* | *T* |  | *rho* | *T* |
| Amygdala | L |  | 0.400 | 3.409 |  | 0.391 | 3.318 |  | 0.285 | 2.324 |
|  | R |  | 0.256 | 2.066 |  | 0.378 | 3.187 |  |  |  |
| Angular gyrus | L |  | 0.542 | 5.039 |  | 0.556 | 5.222 |  | 0.526 | 4.829 |
|  | R |  | 0.509 | 4.618 |  | 0.464 | 4.095 |  | 0.490 | 4.391 |
| Anterior cingulate cortex-pregenual | L |  | 0.380 | 3.205 |  | 0.316 | 2.604 |  | 0.476 | 4.232 |
|  | R |  | 0.256 | 2.070 |  | 0.304 | 2.495 |  |  |  |
| Anterior cingulate cortex-subgenual | L |  |  |  |  | 0.310 | 2.546 |  | 0.306 | 2.511 |
|  | R |  |  |  |  | 0.351 | 2.928 |  | 0.251 | 2.028 |
| Anterior cingulate cortex-supracallosal | L |  | 0.420 | 3.616 |  | 0.346 | 2.881 |  | 0.516 | 4.699 |
|  | R |  | 0.441 | 3.835 |  | 0.326 | 2.697 |  | 0.533 | 4.922 |
| Anterior orbital gyrus | L |  | 0.274 | 2.223 |  |  |  |  | 0.304 | 2.492 |
| Caudate nucleus | L |  | 0.295 | 2.414 |  | 0.408 | 3.487 |  | 0.280 | 2.280 |
| Crus I of the cerebellar hemisphere | L |  | 0.383 | 3.235 |  | 0.337 | 2.796 |  | 0.313 | 2.576 |
|  | R |  | 0.286 | 2.335 |  | 0.364 | 3.049 |  | 0.298 | 2.441 |
| Cuneus | R |  | 0.270 | 2.189 |  | 0.303 | 2.480 |  |  |  |
| Fusiform gyrus | L |  | 0.318 | 2.616 |  | 0.414 | 3.549 |  | 0.291 | 2.373 |
|  | R |  | 0.255 | 2.062 |  | 0.355 | 2.962 |  |  |  |
| Gyrus rectus | R |  |  |  |  | 0.300 | 2.460 |  | 0.327 | 2.703 |
| Heschl’s gyrus | L |  | 0.490 | 4.392 |  | 0.513 | 4.671 |  | 0.353 | 2.942 |
|  | R |  | 0.391 | 3.321 |  | 0.511 | 4.646 |  | 0.340 | 2.822 |
| Hippocampus | L |  | 0.346 | 2.879 |  | 0.493 | 4.431 |  |  |  |
|  | R |  | 0.294 | 2.398 |  | 0.466 | 4.117 |  |  |  |
| IFG pars orbitalis | R |  | 0.253 | 2.043 |  | 0.330 | 2.726 |  |  |  |
| Inferior frontal gyrus-opercular part | L |  | 0.372 | 3.129 |  | 0.294 | 2.402 |  | 0.517 | 4.713 |
|  | R |  | 0.384 | 3.252 |  | 0.290 | 2.363 |  | 0.530 | 4.888 |
| Inferior frontal gyrus-triangular part | L |  | 0.294 | 2.403 |  | 0.277 | 2.250 |  | 0.345 | 2.872 |
|  | R |  | 0.349 | 2.912 |  | 0.271 | 2.200 |  | 0.346 | 2.884 |
| Inferior occipital gyrus | L |  | 0.347 | 2.888 |  | 0.390 | 3.313 |  | 0.341 | 2.836 |
|  | R |  | 0.420 | 3.614 |  | 0.318 | 2.620 |  | 0.413 | 3.542 |
| Inferior parietal gyrus-excluding supramarginal and angular gyri | L |  | 0.496 | 4.458 |  | 0.439 | 3.817 |  | 0.522 | 4.777 |
|  | R |  | 0.405 | 3.458 |  | 0.384 | 3.247 |  | 0.416 | 3.575 |
| Insula | L |  | 0.323 | 2.667 |  | 0.398 | 3.385 |  | 0.340 | 2.820 |
|  | R |  | 0.267 | 2.160 |  | 0.314 | 2.586 |  | 0.337 | 2.792 |
| Lateral orbital gyrus | L |  | 0.402 | 3.432 |  | 0.323 | 2.666 |  | 0.391 | 3.322 |
|  | R |  | 0.382 | 3.226 |  | 0.291 | 2.377 |  | 0.393 | 3.340 |
| Lingual gyrus | L |  | 0.241 | 1.940 |  | 0.371 | 3.118 |  |  |  |
| Lobule IV-V of the cerebellar hemisphere | L |  | 0.344 | 2.857 |  | 0.390 | 3.310 |  | 0.277 | 2.254 |
| Lobule IV-V of vermis |  |  | 0.286 | 2.328 |  | 0.345 | 2.870 |  |  |  |
| Lobule VI of cerebellar hemisphere | L |  | 0.252 | 2.030 |  | 0.282 | 2.292 |  | 0.281 | 2.285 |
|  | R |  | 0.248 | 2.003 |  | 0.425 | 3.670 |  |  |  |
| Medial orbital gyrus | L |  |  |  |  |  |  |  |  |  |
| Mediodorsal medial magnocellular | R |  | 0.407 | 3.477 |  | 0.418 | 3.598 |  | 0.400 | 3.414 |
| Middle cingulate & paracingulate gyri | L |  | 0.416 | 3.573 |  | 0.376 | 3.170 |  | 0.471 | 4.167 |
|  | R |  | 0.433 | 3.748 |  | 0.410 | 3.516 |  | 0.512 | 4.651 |
| Middle frontal gyrus | L |  | 0.392 | 3.326 |  |  |  |  | 0.473 | 4.191 |
|  | R |  | 0.416 | 3.572 |  |  |  |  | 0.430 | 3.723 |
| Middle occipital gyrus | L |  | 0.432 | 3.736 |  | 0.474 | 4.206 |  | 0.360 | 3.018 |
|  | R |  | 0.391 | 3.320 |  | 0.369 | 3.097 |  | 0.386 | 3.268 |
| Middle temporal gyrus | L |  | 0.420 | 3.618 |  | 0.540 | 5.008 |  | 0.358 | 2.998 |
|  | R |  | 0.356 | 2.975 |  | 0.443 | 3.858 |  | 0.280 | 2.278 |
| Olfactory cortex | L |  | 0.290 | 2.364 |  | 0.369 | 3.102 |  | 0.324 | 2.677 |
|  | R |  | 0.287 | 2.338 |  | 0.276 | 2.242 |  | 0.319 | 2.632 |
| Paracentral lobule | L |  | 0.352 | 2.935 |  |  |  |  | 0.491 | 4.399 |
|  | R |  | 0.350 | 2.914 |  | 0.352 | 2.941 |  | 0.414 | 3.552 |
| Parahippocampal gyrus | L |  | 0.376 | 3.172 |  | 0.480 | 4.270 |  | 0.326 | 2.697 |
|  | R |  | 0.346 | 2.882 |  | 0.468 | 4.140 |  | 0.310 | 2.548 |
| Postcentral gyrus | L |  | 0.437 | 3.790 |  | 0.384 | 3.250 |  | 0.418 | 3.595 |
|  | R |  | 0.346 | 2.879 |  | 0.287 | 2.339 |  | 0.339 | 2.811 |
| Posterior cingulate gyrus | L |  | 0.489 | 4.376 |  | 0.475 | 4.214 |  | 0.469 | 4.153 |
| Precentral gyrus | L |  | 0.344 | 2.863 |  |  |  |  | 0.463 | 4.075 |
|  | R |  | 0.286 | 2.331 |  |  |  |  | 0.325 | 2.687 |
| Precuneus | L |  | 0.431 | 3.727 |  | 0.386 | 3.264 |  | 0.342 | 2.845 |
|  | R |  | 0.321 | 2.644 |  | 0.345 | 2.868 |  | 0.289 | 2.361 |
| Pulvinar medial | L |  | 0.277 | 2.253 |  | 0.383 | 3.239 |  |  |  |
| Red nucleus | L |  | 0.293 | 2.391 |  | 0.404 | 3.447 |  |  |  |
| Rolandic operculum | L |  | 0.383 | 3.239 |  | 0.446 | 3.887 |  | 0.281 | 2.285 |
| Substantia nigra-pars compacta | L |  | 0.420 | 3.611 |  | 0.430 | 3.716 |  | 0.306 | 2.507 |
|  | R |  | 0.347 | 2.891 |  | 0.410 | 3.508 |  | 0.280 | 2.281 |
| Substantia nigra-pars reticulata | R |  | 0.319 | 2.629 |  | 0.406 | 3.469 |  | 0.258 | 2.083 |
| Superior frontal gyrus-dorsolateral | L |  | 0.399 | 3.400 |  | 0.258 | 2.087 |  | 0.470 | 4.156 |
|  | R |  | 0.403 | 3.435 |  | 0.276 | 2.241 |  | 0.429 | 3.708 |
| Superior frontal gyrus-medial | L |  | 0.443 | 3.861 |  | 0.260 | 2.104 |  | 0.472 | 4.187 |
|  | R |  | 0.447 | 3.899 |  | 0.273 | 2.218 |  | 0.441 | 3.838 |
| Superior frontal gyrus-medial orbital | L |  | 0.257 | 2.081 |  |  |  |  | 0.304 | 2.492 |
| Superior occipital gyrus | L |  | 0.378 | 3.190 |  | 0.398 | 3.392 |  | 0.252 | 2.037 |
|  | R |  | 0.428 | 3.697 |  | 0.410 | 3.514 |  | 0.332 | 2.748 |
| Superior parietal gyrus | L |  | 0.461 | 4.059 |  | 0.386 | 3.267 |  | 0.451 | 3.948 |
|  | R |  | 0.479 | 4.256 |  | 0.410 | 3.511 |  | 0.403 | 3.435 |
| Superior temporal gyrus | L |  | 0.510 | 4.632 |  | 0.534 | 4.937 |  | 0.380 | 3.213 |
|  | R |  | 0.409 | 3.502 |  | 0.504 | 4.554 |  | 0.282 | 2.291 |
| Supplementary motor area | L |  | 0.400 | 3.407 |  | 0.260 | 2.101 |  | 0.435 | 3.769 |
|  | R |  | 0.398 | 3.390 |  | 0.258 | 2.089 |  | 0.477 | 4.234 |
| SupraMarginal gyrus | L |  | 0.521 | 4.767 |  | 0.530 | 4.883 |  | 0.499 | 4.501 |
|  | R |  | 0.415 | 3.561 |  | 0.412 | 3.535 |  | 0.432 | 3.742 |
| Temporal pole: middle temporal gyrus | L |  |  |  |  | 0.333 | 2.759 |  | 0.252 | 2.031 |
| Temporal pole: superior temporal gyrus | L |  | 0.331 | 2.737 |  | 0.417 | 3.582 |  | 0.356 | 2.971 |
| Mean | |  | 0.369 | 3.138 |  | 0.379 | 3.243 |  | 0.379 | 3.242 |

**(A) MF × FAB, (B) IAF × FAB, (C) SSE × FAB.** Empty cells indicate the regions are not included in the cluster. Regions where age showed potential confounding effects (*i.e.* larger variable importance in projection score in partial least square regression than FAB), were excluded from the list. MF, mean frequency; IAF, individual alpha frequency; SSE, Shannon’s spectral entropy; FAB, Frontal Assessment Battery; *rho*, Spearman’s coefficient; *T*, *T*-statistic
